# Green and applicable chromatographic approaches for the estimation of a multi-component cold and flu relief formulation along with in-vitro dissolution profiling

**DOI:** 10.1038/s41598-026-55497-7

**Published:** 2026-06-10

**Authors:** Mona Nabil, Hoda M. Marzouk, Samah S. Abbas, Hayam M. Lotfy, Nancy W. Nashat

**Affiliations:** 1 Postgraduate program, in Pharmaceutical Analytical Chemistry, Faculty of Pharmacy, Cairo University, Kasr El-Aini Street, Cairo, 11562 Egypt; 2 Pharmaceutical Analytical Chemistry Department, Faculty of Pharmacy, Cairo University, Kasr El-Aini Street, Cairo, 11562 Egypt

**Keywords:** Applicability and sustainability assessment, Chlorpheniramine, Cold & flu pharmaceutical formulation, Dissolution testing, Greenness profile, Ibuprofen, Phenylephrine., Chemistry, Environmental sciences

## Abstract

The emergence of novel pharmaceutical formulations requires the establishment of a reliable analytical method that can accurately quantify active ingredients for use in diverse quality control applications. An over-the-counter pharmaceutical combination of phenylephrine hydrochloride (PHE), chlorpheniramine maleate (CPM), and ibuprofen (IBU) is formulated to treat allergy, lessen fever, and relieve congestion. Two efficient and applicable liquid chromatographic methodologies were established, with an emphasis on ecological sustainability while preserving analytical precision and accuracy, in addition system suitability parameters were successfully determined for each method. The first approach involved high-performance thin-layer chromatography (HPTLC) combined with densitometric quantification, employing silica gel HPTLC 60 F_254_ aluminum sheets as the stationary phase. The developing system comprised ethyl acetate–methanol–aqueous ammonium hydroxide (8.0:2.0:0.1, by volume), and scanning was carried out at 265.0 nm. The respective resolutions (R_s_) were 8.44 and 4.57 for PHE, IBU, and CPM, respectively. The second one is high-performance liquid chromatographic methodology (HPLC), whereas, efficient separation was achieved on a Kromasil 60-5-CN column using isocratic elution of 10.0 mM ammonium acetate buffer and ethanol (50: 50, v/v), adjusted with acetic acid to pH 2.5 at a flow rate of 1.3 mL/min, with DAD quantification at 265.0 nm, with overall run time about 6 min to achieve sufficient separation among the target analytes. The resolutions (Rs) were determined to be 7.62 and 3.21 for PHE, CPM, and IBU, respectively. The investigated approaches’ performance was validated in adherence to the guidelines established by the International Conference on Harmonization guidelines, confirming its reliability for analytical application. Limit of detection (LOD) values were determined to be 0.02, 0.01, and 0.22 µg/band for PHE, CPM, and IBU, respectively in HPTLC method, whilst in HPLC-DAD method, LOD values were 0.03, 0.01, and 0.24 µg/mL for PHE, CPM, and IBU, respectively. These approaches can concurrently estimate the cited drugs in their raw forms, as well as their pharmaceutical combination. In addition, HPLC can monitor their dissolution profiles. Moreover, the applicability profile and ecological sustainability of the studied approaches were verified via employing up-to-date evaluation tools, along with comparisons against official and reported methodologies.

**Supplementary Information:**

The online version contains supplementary material available at 10.1038/s41598-026-55497-7.

## Introduction

Global research is increasingly focused on development of analytical methodologies that prioritize environmental sustainability and minimize adverse impacts on human health and ecosystems. Within this paradigm, the principles of green analytical chemistry (GAC) are recognized as fundamental for environmental preservation and the protection of human health through the mitigation of adverse impacts associated with analytical methodologies^1,2^. Specific assessment tools are mandatory for evaluating the ecological impacts of the suggested approaches. Additionally, the functionality and practicability of the analytical methodologies were assessed using designated and smart applicability evaluation tools. These practicality-focused tools act as a complement to established green assessment matrices. Therefore, a complete evaluation of analytical methods was accomplished via employment of numerous metric tools. The field of chromatography offers a pertinent illustration of these GAC principles in practice.

Chromatography is established as the principal analytical technique in the pharmaceutical industry, acclaimed for its wide availability, high sensitivity, and broad applicability^3–9^. Thin-layer chromatography (TLC) is particularly valued for its cost-effectiveness, simplicity, and rapid analysis. Unlike conventional analytical approaches, TLC effectively achieves the identification, separation, and estimation of the analytes in blends, whether in raw forms, pharmaceutical preparations, or biological fluids, without encountering “memory” impacts. This is consistently achieved via utilizing entirely new stationary phases in all instances. Furthermore, TLC requires minimal sample preparation and reduced solvent volumes, underscoring its economical nature^10–15^.

In parallel, dissolution testing remains a pivotal and interpretative tool in pharmaceutical development, simulating in vivo drug release to verify dosage form performance and ensure product consistency^16–20^. While UV spectrophotometry has been conventionally used for this purpose, it presents significant limitations. These include its restriction to chromophore-containing compounds, susceptibility to spectral overlap in mixtures, insufficient sensitivity and selectivity, and interference from turbid solutions^3,21–23^. Moreover, UHPLC and LC–MS techniques^24,25^, although they are highly advanced and sensitive, but they have some drawbacks such as requiring expensive instrumentation, higher operational cost, and higher energy consumption. As a consequence of these limitations, high-performance liquid chromatography (HPLC) emerged as a powerful, reliable, and attractive alternative, widely applied in biological research and in the pharmaceutical formulations’ dissolution testing. Compared to the commonly used conventional spectrophotometric method, HPLC provides distinct advantages, including superior sensitivity and selectivity, reduced analysis time, compatibility with automation such as laboratory robotics, broad applicability for compound analysis, and high efficiency in large-scale production^3–5^.

Therefore, the simultaneous use of HPTLC and HPLC-DAD was intentionally designed to provide complementary analytical advantages. HPTLC offers a rapid, cost-effective, and high-throughput screening approach with lower solvent consumption and simpler operational requirements, making it suitable for routine quality control and preliminary analysis. In contrast, HPLC-DAD provides higher sensitivity, improved resolution, and superior quantitative accuracy, which are essential for dissolution profiling and precise pharmaceutical analysis. Therefore, the dual-method approach offers flexibility in method selection depending on laboratory resources, analytical requirements, and sustainability considerations.

The over-the-counter (OTC) sector presently constitutes a highly competitive market and a significant focal point of research and development^26^. Additionally, Multicomponent pharmaceutical formulations not only enhance patient compliance by minimizing pill burden but also provide broader therapeutic control over the disease and its associated complications. The combination of phenylephrine, chlorpheniramine, and ibuprofen in a single-dosage form represents a significant category within the over-the-counter (OTC) pharmaceutical market, designed to provide multi-symptom relief from common ailments such as the cold and flu. This formulation leverages a synergistic pharmacological approach: phenylephrine acts as a decongestant to alleviate nasal congestion, chlorpheniramine, an antihistamine, mitigates allergic symptoms like rhinorrhea and sneezing, while ibuprofen, a non-steroidal anti-inflammatory drug (NSAID), addresses associated fever, headaches, and myalgia^27^. The primary significance of such a combination lies in its convenience and accessibility as an OTC medication, enabling self-medication for a complex of co-occurring symptoms without the immediate need for a medical prescription. This not only empowers patients in the management of self-limiting conditions but also potentially reduces the burden on healthcare systems. However, this accessibility necessitates rigorous public education regarding appropriate use, as the inclusion of multiple active ingredients increases the risk of unintended overdosing, drug interactions, and side effects, particularly in vulnerable populations.

**Fig. 1:**
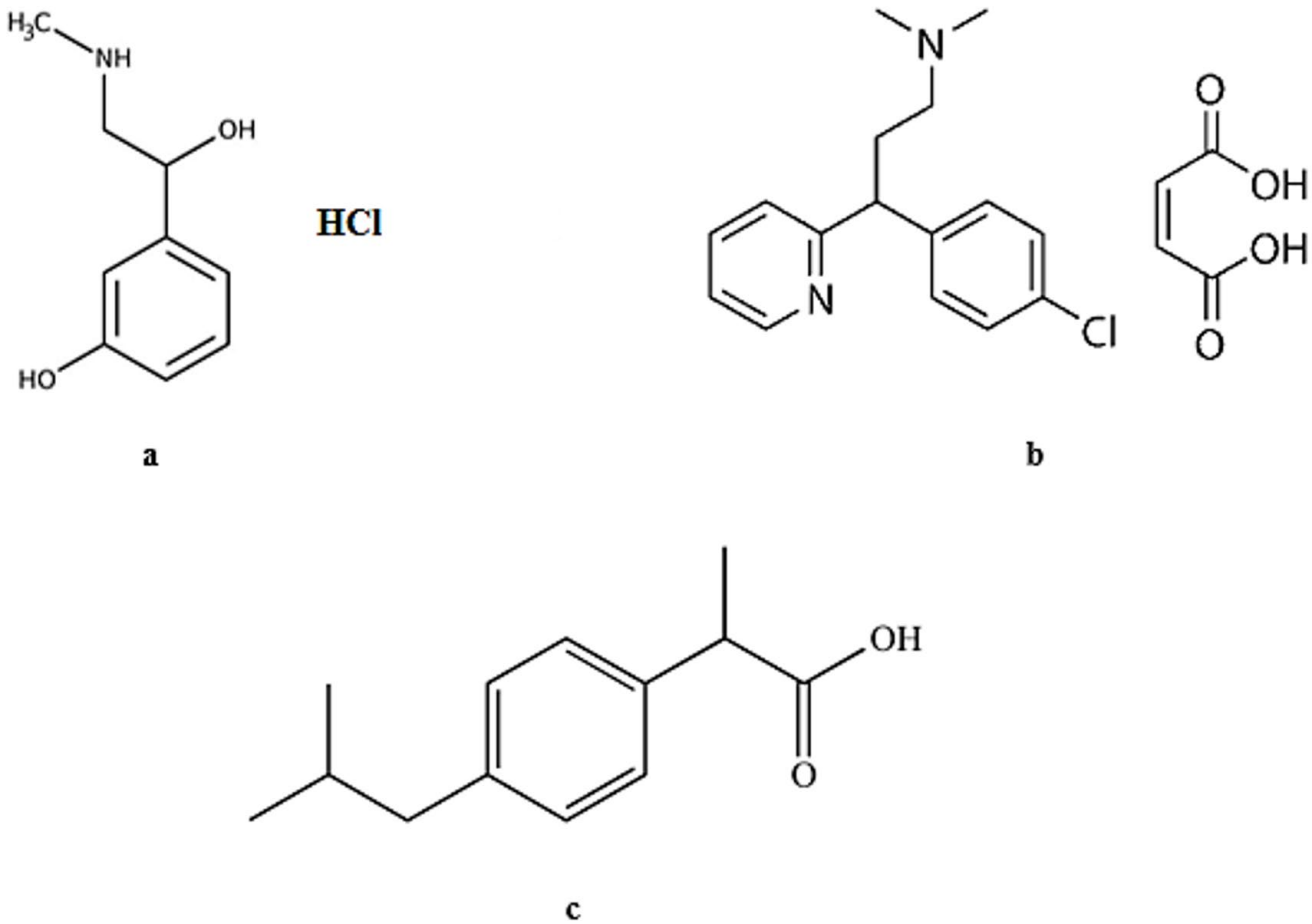
Chemical structures of (**a**) Phenylephrine hydrochloride (PHE), (**b**) Chlorpheniramine maleate (CPM), and (**c**) Ibuprofen (IBU).

Phenylephrine hydrochloride (PHE), hydrogen 3-[(1R)-1-hydroxy-2-(methylamino)ethyl] phenol chloride (Fig. 1a), is as an α₁-adrenergic receptor agonist utilized for the relief of nasal congestion through its pharmacological action^28,29^.

Chlorpheniramine maleate (CPM), (4-Chlorophenyl)-N, N-dimethyl-2-pyridinepropanamine maleate (Fig. 1b), is an antihistaminic and anticholinergic agent extensively employed in pharmaceutical formulations, either as a monotherapy or in combination with other drugs, for the symptomatic management of the common cold and allergic conditions (such as hay fever), often producing a moderate level of sedation^30^.

Ibuprofen (IBU), (RS)-2-(4-(2-methylpropyl) phenyl) propionic acid (Fig. 1c), is a nonsteroidal anti-inflammatory drug (NSAID) that acts as cyclooxygenase inhibitor utilized as an analgesic in the management of fever and arthritis^31^. Advil Allergy & Congestion relief caplets alleviate multiple sinus and allergy symptoms in a single dose by combining the effectiveness of the three cited drugs in the ratio of PHE: CPM: IBU (2.5: 1: 50)^32,33^.

A review of the literature indicates that PHE, CPM, and IBU in caplet dosage forms have been concurrently quantified utilizing HPLC^34–37^ methods. The objective of this study is to develop rapid and eco-friendly chromatographic methods for the separation of PHE, CPM, and IBU in their challenging ratio (2.5: 1: 50). These methods aim to be easily integrated into routine quality control processes. Given that this mixture is commonly used over-the-counter for multi-symptom cold and flu relief, the need for precise and reliable quality control measures is especially critical to ensure product safety and efficacy. Furthermore, the method’s applicability is particularly relevant for drugs that are not liable to degradation under typical working conditions. As a result, the method provides a reliable and efficient solution for quality control purposes.

Our primary aim in this study is to present an effective, economic, robust, applicable, and ecologically sustainable HPTLC- densitometric and HPLC-DAD approaches to concurrently quantify the studied components in both their bulk forms and pharmaceutical formulations, achieving superior resolution with optimized run time. In addition, an effective in vitro dissolution monitoring method was established for the Advil Allergy & Congestion relief caplets utilizing the proposed HPLC-DAD approach according with FDA specifications and USP recommendations^18,38^. The previous approaches are limited by relatively high solvent consumption, longer analysis time, complex mobile phase composition, and limited application in dissolution profiling, and insufficient sustainability and applicability assessment, which reduce their greenness and routine applicability in quality control laboratories. These limitations restrict their applicability in routine quality control and green analytical chemistry frameworks. Therefore, the novelty and applicability of the proposed chromatographic methods lie in the development of optimized HPTLC and HPLC-DAD approaches that provide rapid separation in short run time, reduced solvent consumption, improved selectivity, and reliable application to dissolution profiling studies, and integration of novel and innovative green, applicability, and sustainability assessment tools. Moreover, applicability profile, estimation of CO_2_ emission, and ecological sustainability of the recommended approaches were guaranteed, and compared among reported approaches. These tools were consequently employed to the reported methodologies which were employed to concurrently analyze the target analytes^34–37^.

## Experimental

### Instrumentation

#### For HPTLC- densitometry

HPTLC system comprises a CAMAG Linomat 5 autosampler made by CAMAG in Muttenz, Switzerland coupled with 100 µL CAMAG microsyringe. A CAMAG TLC scanner from the model 3 S/N 1,302,139 was utilized for scanning, together with WinCATS software for densitometric evaluation. The plates were visualized utilizing a UV lamp with a wavelength of 265.0 nm. Silica gel HPTLC 60 F_254_ aluminum plates, measuring 20 × 20 cm and 0.25 mm in thickness were purchased from Merck (Darmstadt, Germany). The measurement mode employed was reflectance, with a slit dimension of 3 × 0.45 mm and a scanning speed of 20 mm/s. The output comprised a chromatogram and integrated peak area.

#### For HPLC- DAD

The Waters Alliance HPLC system (Waters Corporation, Milford, MA, USA) was employed, consisting of a 2690 series quaternary pump with variable flow rate capability, integrated with a Waters 996 photodiode array UV detector was operated at a bandwidth of 4 nm with a spectral scanning range of 200–400 nm, and scanning was carried out at 265.0 nm, a vacuum degasser, and an autosampler equipped with a 100-µL injection loop. Data acquisition, processing, and reporting were conducted using Empower software (Waters Corporation, Milford, MA, USA). System suitability parameters were performed before each analytical run to ensure proper system performance. The evaluated parameters included retention time stability, peak symmetry, theoretical plate count, and resolution.

The Vankel VK 7000 device (Varian, Inc., now part of Agilent Technologies, USA), equipped with six vessels and a standard USP type-II paddle, was used for in vitro dissolution monitoring of Advil Allergy & Congestion relief caplets.

### Materials and reagents

Ethanol and ammonium acetate of HPLC-grade, and analytical-grade reagents such as **e**thyl acetate, methanol, acetic acid, and ammonia solution (33% w/w) were purchased from Merck (Darmstadt, Germany). Double distilled deionized water was acquired from Sigma Aldrich Chemie (Darmstadt, Germany).

### Samples

Phenylephrine (PHE), chlorpheniramine (CPM), and Ibuprofen (IBU) reference standards were attained from Global NAPI Pharmaceutical Industries (Cairo, Egypt). The purities were affirmed as 100.24% ± 0.70 for PHE, 100.10% ± 0.64 for CPM, and 100.01% ± 0.51 for IBU, based on their respective official methods^39^. Advil Allergy & Congestion relief caplets (Lot No. 070140 A) were made by Haleon (New Jersey, USA). Each unit dose is formulated to contain 10.0 mg of PHE, 4.0 mg of CPM, and 200.0 mg of IBU.

### Standard solutions

Standard stock solutions of PHE, CPM, and IBU were each made at 1.0 mg/mL by accurately weighing 100.0 mg of each analyte, dissolving in ethanol as the solvent, and diluting to volume in 100-mL volumetric flasks. In addition, PHE, CPM, and IBU working solutions (100.0 µg/mL) were prepared via proper dilution of their respective standard solutions in methanol for HPTLC analysis and in the mobile phase for HPLC analysis.

### Procedure

#### Chromatographic conditions

##### For HPTLC – densitometry

The analysis process was conducted on 20 × 10 cm HPTLC aluminum sheets that were precoated with silica gel F_254_. Volumes of 10 µL were applied in triplicate from various solutions’ concentrations as distinct compact bands, each separated by 10 mm from one another, and positioned 10 mm from the plates’ bottom edges and sides, with each band measuring 6 mm in length. In a pre-saturated chromatographic tank, a linear ascending development was executed at room temperature for approximately 30 min utilizing a developing system encompassing ethyl acetate, methanol, and ammonia solution (8.0:2.0:0.1, by volume). The plates underwent development over a linear distance of 8 cm, followed by subsequent air - drying at ambient temperature. The isolated chromatographic bands were visualized and detected utilizing a UV lamp and subsequently scanned at a wavelength of 265 nm.

##### For HPLC – DAD

Chromatographic separation was carried out at ambient temperature using a Kromasil 60-5-CN column (250 × 4.6 mm, 5 μm) was made by Nouryon in Sweden, under isocratic elution mode with 10 mM ammonium acetate buffer: ethanol (50:50, v/v), adjusted with acetic acid to pH 2.5. The mobile phase was passed through a Millipore membrane filter (0.45 μm) prior to being pumped at a flow rate of 1.3 mL/min. A 250 µL analytical syringe was utilized to inject a volume of 10 µL. The eluent was monitored at 265.0 nm using a diode array detector (DAD). All measurements were performed at 25° C.

#### Calibration curves’ construction

##### For HPTLC – densitometry

Precise volumes equivalent to 1.0–140.0 µg for PHE, 1.0–100.0 µg for CPM, and 20.0–400.0 µg for IBU, were precisely transferred from their respective standard solutions (1.0 mg/mL) into four sets of 10- mL volumetric flasks. The volumes were completed to the mark utilizing methanol, resulting in final concentrations of 0.1–14.0 µg/band for PHE, 0.1–10.0 µg/band for CPM, and 2.0–40.0 µg/band for IBU. The above- mentioned chromatographic conditions were implemented, followed by recording the peak areas. Calibration curves correlating the respective peak areas with the equivalent concentrations were subsequently generated, and the regression equations were computed.

##### For HPLC-DAD

Aliquots containing concentrations ranging from 2.0 to 240.0 µg for PHE, 1.0–100.0 µg for CPM, and 20.0–3500.0 µg for IBU, were precisely transferred from their respective 1.0 mg/mL stock solutions into four separate 10-mL volumetric flasks. The flasks were completed to the final volume with the mobile phase to achieve final concentrations of 0.2–24.0 µg/mL for PHE, 0.1–10.0 µg/mL for CPM, and 2.0–350.0 µg/ mL for IBU. The rationale for extension of calibration range of IBU to 350.0 µg is due to its ratio in dosage form; PHE: CPM: IBU (2.5:1:50), as it is the component with highest concentration in dosage form. The above-described chromatographic conditions were executed in triplicate, and the resulting chromatograms were recorded. Calibration curves were constructed by correlating the average peak areas with their respective concentrations, followed by the calculation of the regression equation for each analyte.

#### Laboratory prepared mixtures’ assay

The mentioned approaches’ selectivity was ensured through analysis of laboratory prepared mixtures encompassing varying ratios of the target drugs in their bulk materials. Proper portions of pure PHE, CPM, and IBU were precisely transferred from their particular standard solutions (1.0 mg/mL) into three sets of 10- mL volumetric flasks, and the volumes were accomplished to the calibration mark with methanol (for HPTLC) and mobile phase (for HPLC). The above - specified chromatographic conditions were implemented, and the consequent regression equations were subsequently utilized to determine the analyte concentrations.

#### Application to pharmaceutical formulation

Ten Advil allergy & sinus relief caplets were each weighed, then crushed and mixed. An amount equal to one caplet was put into a 100-mL beaker, 50.0 mL of ethanol was added, and the mixture was sonicated for 30 min. Moreover, the sonication was performed to ensure complete dissolution without affecting analyte integrity. The resulting solution was filtered into a 100-mL volumetric flask, and the volume was adjusted to the mark utilizing ethanol as the diluent. The resultant solution was subsequently diluted with methanol for HPTLC analysis and with the mobile phase for HPLC analysis, thereby attaining concentrations within the established linearity ranges of PHE, CPM, and IBU. The analytical procedures were then executed in accordance with each approach. The eventual concentrations attained for HPTLC approach were1.0 µg/band for PHE, 0.4 µg/band for CPM, and 20.0 µg/band for IBU, and the recovery percentages were determined to be 100.33 ± 0.58 for PHE, 100.93 ± 0.98 for CPM, and 99.18 ± 0.26 for IBU, whilst the concentrations achieved for HPLC-DAD approach were 5.0 µg/band for PHE, 2.0 µg/band for CPM, and 100.0 µg/band for IBU, with recovery percentages of 100.87 ± 0.31, 99.70 ± 0.70, 100.16 ± 0.62 for PHE, CPM, and IBU, respectively. Moreover, the accuracy of the proposed method was verified via application of standard addition technique, which involves the addition of pure standard drugs to the respective pharmaceutical preparation.

#### Dissolution profiling

The previously described HPLC-DAD method was employed to monitor the in-vitro dissolution profiles of Advil allergy & sinus relief caplets in 50 mM potassium phosphate buffer with pH 6.5, in accordance with FDA specifications^18^.The dissolution volume (900 mL) maintained sink conditions throughout the experiment, as the maximum theoretical concentration of IBU (200 mg/900 mL ≈ 0.22 mg/mL) is well below its solubility in pH 6.5 buffer (reported > 10 mg/mL). Dissolution kinetics were executed via utilizing a USP type II dissolution apparatus, wherein a single caplet was introduced into a vessel containing 900 mL of 50 mM potassium phosphate buffer with pH 6.5 as the dissolution medium. The dissolution system was adjusted at a controlled temperature of 37 ± 0.5 °C and operated at an agitation speed of 50 rpm. At designated time intervals of 5.0, 10.0, 15.0, 20.0, and 30.0 min, 5.0 mL samples were withdrawn, followed by substitution of the volume with an equal volume of fresh medium. After collection, the samples were filtered through a syringe filter possessing a pore size of 0.22 μm. The experiment was performed in triplicate. The dissolution percentage was computed by applying each analyte’s respective regression equations in the chosen medium, after which the in-vitro release profiles of PHE, CPM, and IBU were constructed. Proper dilutions of PHE, CPM, and IBU standard solutions were prepared individually into three separate sets of 10- mL volumetric flasks, using 50.0 mM potassium phosphate buffer with pH 6.5 as a solvent. Calibration curves were then established, and each analyte’s respective regression parameters were computed.

## Results and discussion

The imperative to ensure pharmaceutical safety and quality demands rigorous analytical control throughout manufacturing. It is for this reason that chromatography serves as the preeminent technique for routine drug analysis in quality control laboratories globally. TLC is the most frequently employed technique among the diverse planar chromatographic techniques for the isolation and quantitation of the components, primarily due to its affordability and operational simplicity. TLC was improved by the emergence of HPTLC, which involves a significant reduction in the particle size of stationary phases and the thickness of the plates. The elevated separation efficiency attained, as indicated by well- resolved and narrow bands in the chromatogram, supports the utilization of this methodology for the detection and isolation of the analytes^10–15^. The traditional (ordinary LC) has been extensively employed in separation and estimation of the analytes owing to its pronounced sensitivity and economically favorable separation capabilities. The availability of diverse stationary phases and detectors has further expanded its applicability. HPLC-DAD methodology has emerged as a prominent, robust, and reliable tool in scientific research, serving as an effective alternative for biological studies and dissolution testing of pharmaceutical formulations. In addition, HPLC presents distinct advantages, encompassing superior selectivity and sensitivity, reduced analytical time, suitability for automation, broad applicability for the analysis of diverse compounds, and high efficiency for large-scale pharmaceutical production^3–5^. The complementary nature of the two developed methods warrants explicit discussion. The HPTLC-densitometric method is particularly advantageous for laboratories with limited budgets or instrumentation, as it requires no high pressure pumps or expensive columns, allows simultaneous analysis of up to 20 samples on a single plate, and eliminates carryover effects by using fresh stationary phase for each run. Its operational simplicity and low cost per sample make it ideal for routine batch release testing in small scale quality control environments. Conversely, the HPLC-DAD method provides greater sensitivity (LOD values down to 0.01 µg/mL for CPM), higher resolution (Rs > 3.2 for all peaks), and fully automated sequential analysis. These features are essential for dissolution profiling, where multiple time points must be analyzed precisely and reproducibly. Furthermore, the HPLC method can be readily integrated into automated dissolution workstations, whereas the HPTLC method would require manual sample application for each time point. Thus, rather than being redundant, the two methods address distinct analytical scenarios and together offer a comprehensive toolkit for pharmaceutical analysis. Therefore, this research’s primary goal is to establish a reliable, robust, selective, applicable, and ecologically sustainable approaches for determination of phenylephrine (PHE), chlorpheniramine (CPM), and ibuprofen (IBU). The assessment of the mentioned analytes’ dissolution profiles was accomplished via utilizing the studied HPLC-DAD approach. Ultimately, the studied approaches’ applicability and health implications were ensured via diverse applicability and greenness assessment tools.

### Optimization of chromatographic conditions

The choice of the optimum conditions for the proposed chromatographic methods depends on factors such as the nature of the drugs, the complexity of the sample, and the intended use. In this study the conditions were influenced by the physical-chemical properties of phenylephrine hydrochloride (PHE), chlorpheniramine maleate (CPM), and ibuprofen (IBU), such as solubility, polarity, UV absorption, and interference. The apparent pKa of (PHE) has two pK_a_ 9.69 and 9.06 (~ 9.4), while pK_a_ of CPM, and IBU were 9.2, and 4.9, respectively. The strategic use of minimal amounts of ethanol and methanol in these analytical procedures offers several advantages aligned with principles of green chemistry. These solvents are comparatively less toxic than many traditional organic solvents, thereby reducing health risks for analysts and decreasing the need for extensive safety measures. Environmentally, the reduced solvent volumes lead to decreased chemical waste and pollution, given that both ethanol and methanol are biodegradable and exhibit lower persistence in the environment. Cost-wise, smaller solvent quantities diminish expenses associated with purchasing, storage, and disposal. This approach effectively balances the need for analytical efficiency with environmental sustainability, serving as a compromise that maintains analytical performance while minimizing ecological impact.

#### For HPTLC- densitometry

The influence of multiple experimental parameters on the performance of the studied HPTLC-densitometric method was investigated to achieve its optimization. The optimization of the mentioned approach was accomplished, with emphasis on utilizing ecologically sustainable solvents, whilst omitting the hazardous alternatives such as chloroform, benzene, toluene, acetonitrile. Initial trials were conducted applying diverse environmentally benign developing systems, including water-ethanol, ethyl acetate-butanol, ethyl acetate–ethanol, and ethyl acetate–methanol, prepared in various ratios (3:7, 4:6, 5:5, 6:4, 7:3, and 9:1, by volume). Nevertheless, the outcomes were deemed unsatisfactory owing to pronounced peak tailing (> 2), and insufficient resolutions (< 1.5) among the analytes under investigation, below acceptable limit. Upon utilizing ethyl acetate-methanol (8:2, by volume) as a developing system, separation of the target analytes was attained, but poor resolution, broad peaks, and pronounced peak tailing were detected. Therefore, diverse pH modifiers to the developing system of ethyl acetate-methanol were tried such as triethylamine, glacial acetic acid, and aqueous ammonium hydroxide. CPM exhibited baseline retention under acidic pH conditions. Conversely, better separation was achieved when employing an alkaline pH in the developing mixture. Aqueous ammonium hydroxide was favored over triethylamine owing to its reduced environmental impact and its capacity to yield superior band symmetry. Various volumes of aqueous ammonium hydroxide were investigated, and the optimum ammonia ratio was determined to be (0.1, by volume), providing the best balance between resolution and band symmetry. An increase in the proportion of aqueous ammonium hydroxide resulted in greater retention of IBU at the baseline. Ultimately, complete separation of all eluted compounds, exhibiting proper retardation factor Rf values of 0.25 ± 0.01, 0.63 ± 0.02, and 0.79 ± 0.02 for PHE, IBU, and CPM, respectively along with sharp and symmetric peaks, was achieved utilizing a developing system composed of ethyl acetate –methanol – aqueous ammonium hydroxide (8.0: 2.0 :0.1, by volume) (Fig. 3a). Methanol was chosen due to excellent chromatographic properties, eco-friendliness and compatibility with the target analytes to provide superior resolution. This separation agrees with the analytes properties at basic developing system since Phenylephrine hydrochloride (PHE), a polar compound in basic phase with high pK_a_ (~ 9.4), remains un-protonated at basic pH, resulting in strong hydrogen bonding and low mobility, reflected by its low R_f_ of approximately 0.25. Ibuprofen (IBU), with a moderate polarity and a pK_a_ of around 4.9, becomes negatively charged at basic pH, increasing its solubility in the developing system, leading to an intermediate R_f_ of about 0.63. Chlorpheniramine (CPM), a less polar, lipophilic compound with a pKa 9.2, remains unprotonated and more hydrophobic at basic pH, exhibiting weak interactions with the stationary phase and a high R_f_ of approximately 0.79. Overall, the R_f_ order is PHE < IBU < CPM, corresponding to their polarity and interactions with the stationary and developing system. Considering the ratio of the studied drugs in the dosage form, PHE: CPM: IBU (2.5: 1: 50). Multiple detection wavelengths (210.0, 254.0, 265.0, and 275.9 nm) were systematically evaluated for densitometric analysis, with consideration given to the absorbance spectra of the analytes under investigation. Scanning at 265.0 nm yielded the highest sensitivity for CPM, as it is the λ _max_ of CPM which is present as the component of low concentration at dosage form as well as acceptable results for PHE, and IBU. Thus, the wavelength of 265.0 nm was selected to be the optimal wavelength, as it achieved highest sensitivity and superior peak symmetry for all drugs, in addition to displaying minimal baseline noise, as illustrated in Fig. 3a.

**Fig. 2:**
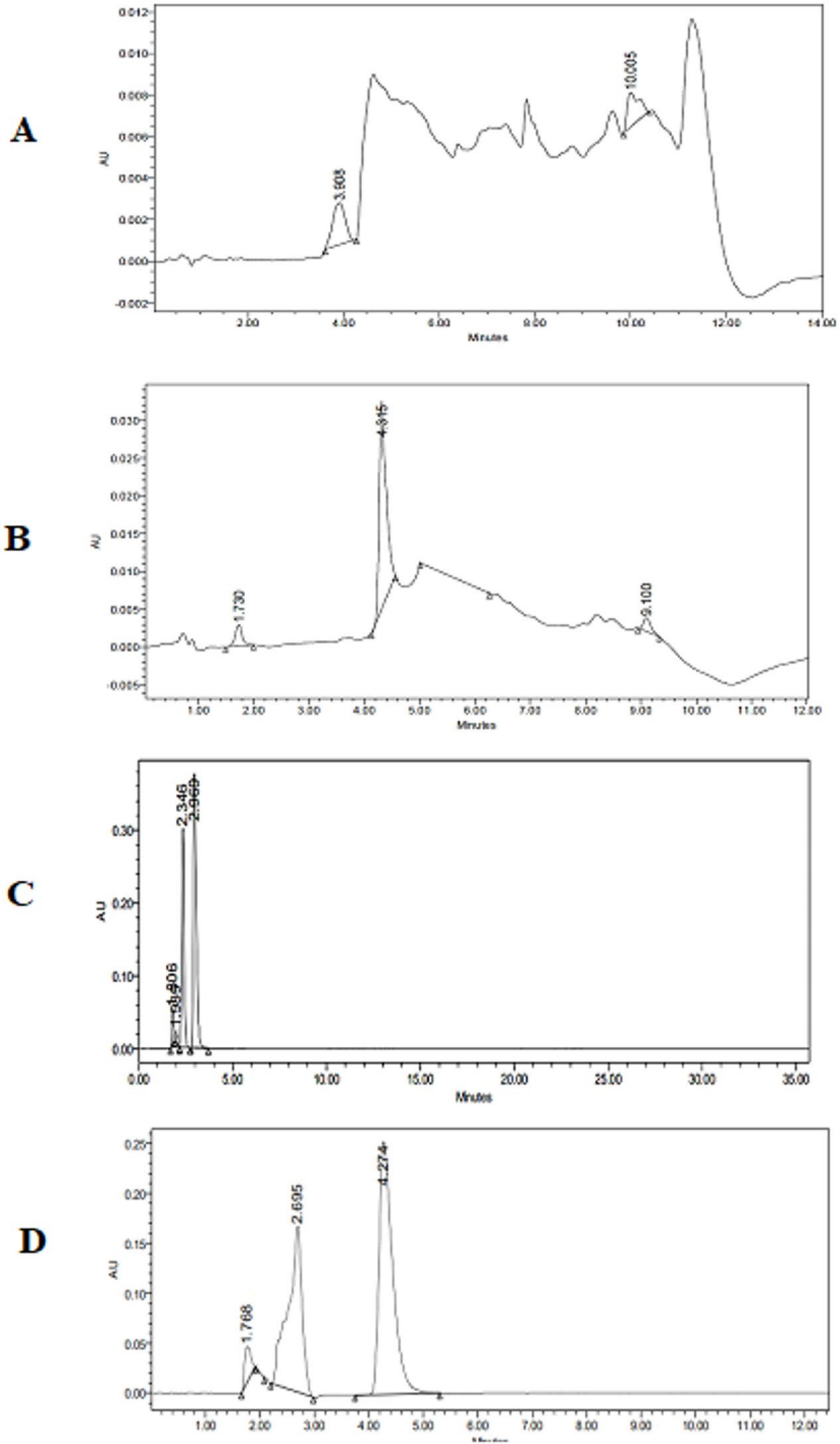
HPLC chromatograms for trials utilizing isocratic elution of 10 mM ammonium acetate buffer and ethanol, adjusted to pH 2.5; (**A**) (90: 10, v/v), (**B**) (10: 90, v/v), (**C**) (80: 20, v/v), and (**D**) (70: 30, v/v).

**Fig. 3:**
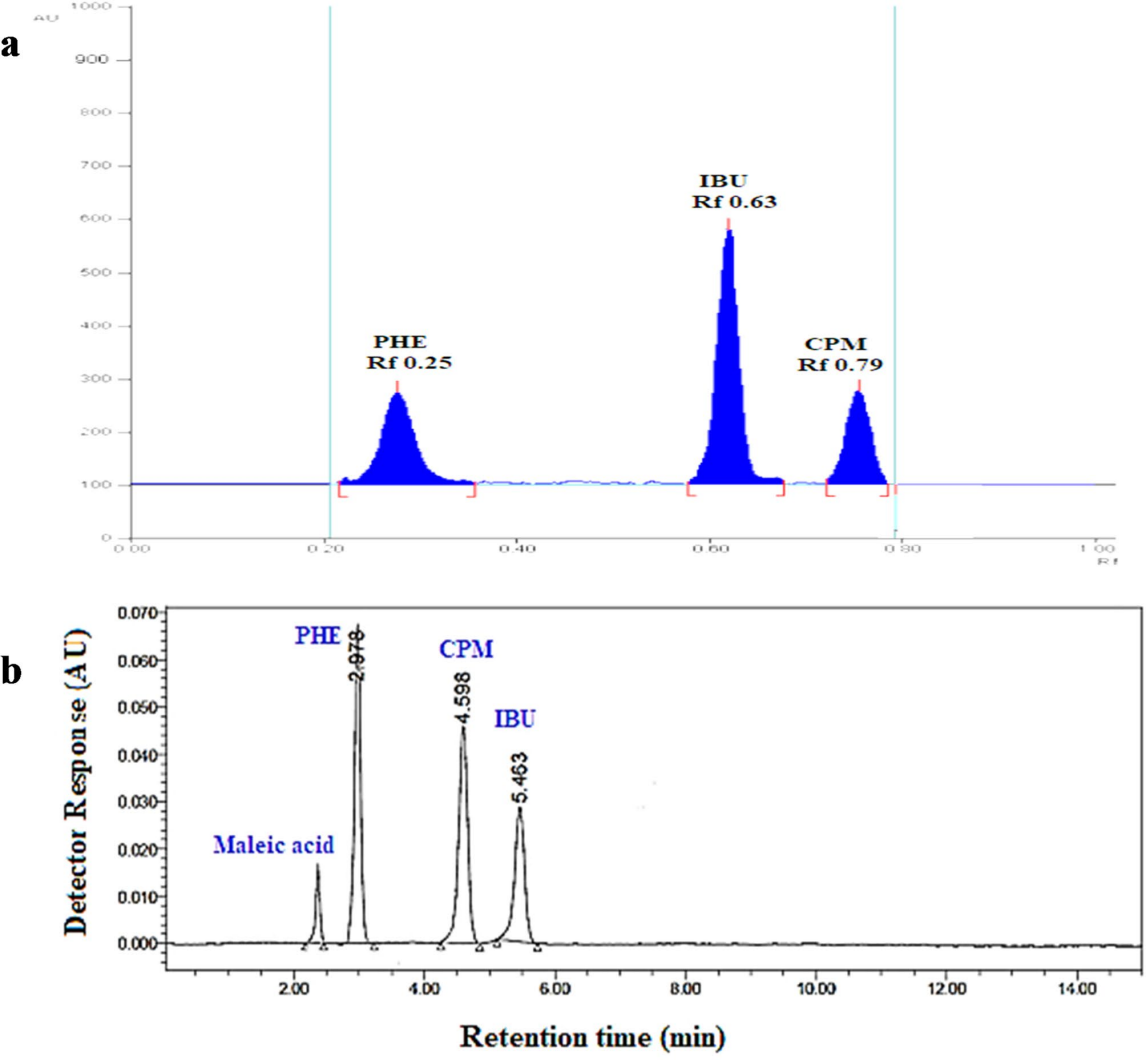
**(a)** HPTLC-densitogram of a mixture of PHE (2.0 µg/band), IBU (40.0 µg/band), and CPM (0.8 µg/band), scanned at 265.0 nm using ethyl acetate – methanol - aqueous ammonium hydroxide (8.0: 2.0: 0.1, by volume) as the developing system and (**b**) HPLC-DAD chromatogram of a mixture of PHE (5.0 µg/mL), CPM (2.0 µg/mL), and IBU (100.0 µg/mL) using isocratic elution of 10 mM ammonium acetate buffer and ethanol (50: 50, v/v), adjusted with acetic acid to pH 2.5 at 265.0 nm.

#### For HPLC- DAD

Diverse parameters were evaluated to enhance the optimization of the studied HPLC-DAD approach. Various organic solvents, such as acetonitrile, methanol, and ethanol, along with aqueous phases including water, acetate buffer, phosphate buffer, and 0.1% formic acid, were investigated with various ratios, flow rates, and pH conditions. Acetonitrile was omitted to promote the ecological sustainability of the approach. Ethanol was chosen, but not only for its Eco-friendly profile but also for its favorable chromatographic properties. Compared to methanol and acetonitrile, ethanol exhibits moderate eluotropic strength and higher viscosity, which slows down analyte elution and enhances interaction with the stationary phase, leading to improved selectivity and peak resolution and shape. Additionally, ethanol has different hydrogen-bonding and polarity characteristics that influence solute–mobile phase–stationary phase interactions, resulting in better separation of the studied analytes. In contrast, acetonitrile showed stronger elution power and faster elution, which reduced retention and insufficient resolution, whilst methanol produced broader peaks and less satisfactory selectivity under the tested conditions. Ethanol also provided a stable baseline, acceptable retention times, sufficient resolutions, and satisfactory system suitability parameters, confirming its chromatographic suitability. Therefore, ethanol was selected based on both chromatographic performance and green chemistry considerations.

During the method development phase, alternative greener solvent systems, including ethanol-water mixtures and fully aqueous buffers, were evaluated as potential substitutes for the traditional organic solvents. However, these alternatives resulted in compromised chromatographic performance, manifested by reduced resolution (Rs < 1.5), broadened peak shapes, and diminished sensitivity towards the target analyte. Consequently, the selected solvent systems were optimized to ensure analytical robustness and efficiency. To this end, a series of solvent mixtures, comprising water, 0.1% formic acid, and phosphate buffer at pH values of 3.0, 4.0, and 5.0, in various ratios with ethanol, were investigated. Regrettably, these combinations were found to be suboptimal, yielding poor resolution among the target analytes (Rs < 1.5) and delayed elution of IBU (> 14 min).

Despite encountering these difficulties, comprehensive optimization of the mobile phase composition was conducted, involving the implementation of various isocratic programs. Ammonium acetate buffer is regarded as more environmentally friendly than traditional alternatives such as phosphate buffers because it is less toxic, biodegradable, and produces fewer disposal concerns. Phosphate buffers can pose environmental issues due to their potential to contribute to eutrophication and their more complex disposal requirements. In contrast, ammonium acetate decomposes more readily and has a lower ecological footprint. Ethanol is a renewable, biomass-derived solvent that aligns with green chemistry principles, reducing reliance on hazardous organic solvents like acetonitrile or methanol. Using ethanol, which is less toxic and more environmentally benign, supports the development of greener analytical procedures. Binary solvent mixtures consisting of ethanol and methanol, each combined with 10 mM ammonium acetate buffer at pH values of 4.0, 5.0, and 6.0, in a range of ratios (20:80, 30:70, 50:50, 70:30, and 80:20, v/v), were investigated. However, the ensuing results revealed inadequate chromatographic performance, marked by low resolution (Rs < 1.5), below the acceptable limit, as well as peak broadening and tailing, with tailing factors exceeding the acceptable threshold (> 2); Fig. 2. The best compromise among peak shapes, adequate resolution, and acceptable retention times was achieved utilizing isocratic elution of 10 mM ammonium acetate buffer and ethanol (50:50, v/v), adjusted to pH 2.5 with acetic acid with retention time is 2.97 ± 0.03 min for PHE, 4.59 ± 0.02 min for CPM, and 5.46 ± 0.03 min for IBU; Fig. 3b .This combination ratio of ammonium acetate with ethanol (50:50, v/v) enhances the sustainability of the analytical method.

Recent green chromatography literature emphasizes that sustainability is best achieved by reducing analysis time, minimizing solvent volume per run, and employing less hazardous mobile phases—all principles incorporated into this method. Thus, the use of ethanol and ammonium acetate buffer with moderate flow rates and short run times exemplifies green analytical chemistry by reducing total solvent consumption and employing environmentally friendly reagents, without sacrificing analytical performance. This approach demonstrates a balanced strategy to achieve effective separation while adhering to sustainability goals. The higher viscosity of ethanol compared to conventional organic solvents can impact chromatographic performance by increasing system backpressure and potentially reducing column efficiency. To address this, the method employed a moderate flow rate of 1.3 mL/min, which balanced analysis time, resolution, and system pressure. The analysis time was kept short (~ 6 min), and the total solvent volume per run was only 7.8 mL (calculated as flow rate × run time), minimizing solvent consumption.

Despite ethanol’s viscosity, these optimized conditions allowed the system to operate within acceptable pressure limits, achieving satisfactory resolution and peak shapes, with no compromise on analyte stability under the acidic conditions used. The method utilized flow rates of 1.0, 1.3, and 2.0 mL/min, with 1.3 mL/min providing the best trade-off between speed and efficiency.

Moreover, in the above- mentioned isocratic programs, multiple stationary phases were tried, namely Kromasil 60-5- CN column, YMC Triart-Phenyl, YMC C_8_, and YMC C_18_ columns. Upon utilizing YMC Triart-Phenyl (150 × 4.6 mm, 5 μm), and YMC C_8_ column (100 × 4.6 mm, 5 μm), suboptimal chromatographic performance was observed, as the resolution among the analytes was < 1.5, below the acceptable criterion, while the peaks exhibited significant broadening with a tailing factor exceeding 2. Whilst, utilization of YMC C_18_ column (150 × 4.6 mm, 5 μm) resulted in pronounced retention of IBU, with a retention time of approximately 9 min, thereby extending the overall run time, and a significant peak tailing was observed, as indicated by a tailing factor exceeding 2. The Kromasil 60-5- CN column (250 × 4.6 mm, 5 μm) is a cyano-bonded silica column with moderate polarity. The CN phase provides a balanced combination of polar and hydrophobic interactions, making it well-suited for analyzing compounds with a wide range of polarities. It facilitates faster elution of polar analytes while effectively retaining moderately hydrophobic molecules, thereby enabling efficient and versatile separation of diverse compounds with retention times of 2.97 ± 0.03 min for PHE, 4.59 ± 0.02 min for CPM, and 5.46 ± 0.03 min for IBU, respectively. Thus, this column demonstrated superior performance, providing the highest resolution, proper peak shapes, and optimal system suitability parameters, with resolution values exceeding 2 for the analytes under investigation, a selectivity factor greater than 1 (1.1 indicating optimal separation), a tailing factor below 2, and column efficiency exceeding 2000 theoretical plates, as illustrated in Fig. 3b. Moreover, this column was selected owing to its distinct chemical purity, chemical stability, superior durability, and defined pore structure, and its capability to deliver enhanced resolution and peak symmetry with negligible tailing. The pK_a_ values of the drugs significantly influence their ionization states and chromatographic behavior at pH 2.5. This separation order due to that phenylephrine hydrochloride (PHE), with a pK_a_ of approximately 9.4 for its basic amine group, is fully protonated and positively charged at this pH, resulting in high polarity and a short retention time of about 2.97 min due to weak interactions with the hydrophobic stationary phase. Chlorpheniramine maleate (CPM), with a pK_a_ near 9.2, is also fully protonated under these conditions but possesses aromatic and alkyl groups that increase its lipophilicity, leading to a moderate retention time of around 4.59 min through stronger hydrophobic interactions. Ibuprofen (IBU), with a lower pKa of approximately 4.9 for its carboxylic acid, remains largely non-ionized and neutral at pH 2.5, which enhances its hydrophobicity and results in the longest retention time of about 5.46 min due to robust interactions with the stationary phase. The increasing order of retention PHE < CPM < IBU—correlates with their polarity and hydrophobicity, and resolution values exceeding 2 confirm effective baseline separation for accurate quantification. Moreover, regarding the Kromasil 60-5- CN column lifetime and repeatability, the use of low volume of benign solvent content, moderate flow rate, short run time and controlled pH conditions (pH 2.5) which lies within the commonly recommended operational range for silica-based columns (typically pH 2.0 − 8.0) is expected to prolong the cyano -column lifetime, minimize potential column degradation and maintain consistent chromatographic performance. Moreover, Reproducibility across instruments is supported by the use of standard HPLC operating parameters, making the method transferable to conventional chromatographic systems.

The choice of the wavelength range for HPLC UV-VIS analysis is a critical factor influencing the approach’s analytical performance. This choice directly impacts critical analytical parameters, including selectivity, sensitivity, linearity, and overall method stability. Regarding the ratio of the proposed drugs in the dosage form, PHE: CPM: IBU (2.5: 1: 50). A diode array detector was employed, and various detection wavelengths (210.0, 254.0, 265.0, and 275.9 nm) were examined. Meanwhile, the wavelength of 265.0 nm gave the highest sensitivity for CPM, as it is the λ _max_ of CPM which is present as the component of low concentration at dosage form as well as satisfactory results for PHE, and IBU. Therefore, the wavelength of 265.0 nm was determined to be the most appropriate, as it achieved superior sensitivity and peak symmetry for all analytes, in addition to exhibiting minimal baseline noise. Ultimately, the chromatographic optimization was performed via investigation of each variable separately, including mobile phase composition (solvent type, ratio, pH), Stationary phase (CN, C_18_, C_8_, phenyl), Flow rate and detection wavelength.

### System suitability

The chromatographic approaches’ performance was verified via determining system suitability parameters in compliance with USP guidelines^38^, which serve as critical indicators of their analytical reliability.

#### Suitability parameters for HPTLC-densitometry

**Fig. 4:**
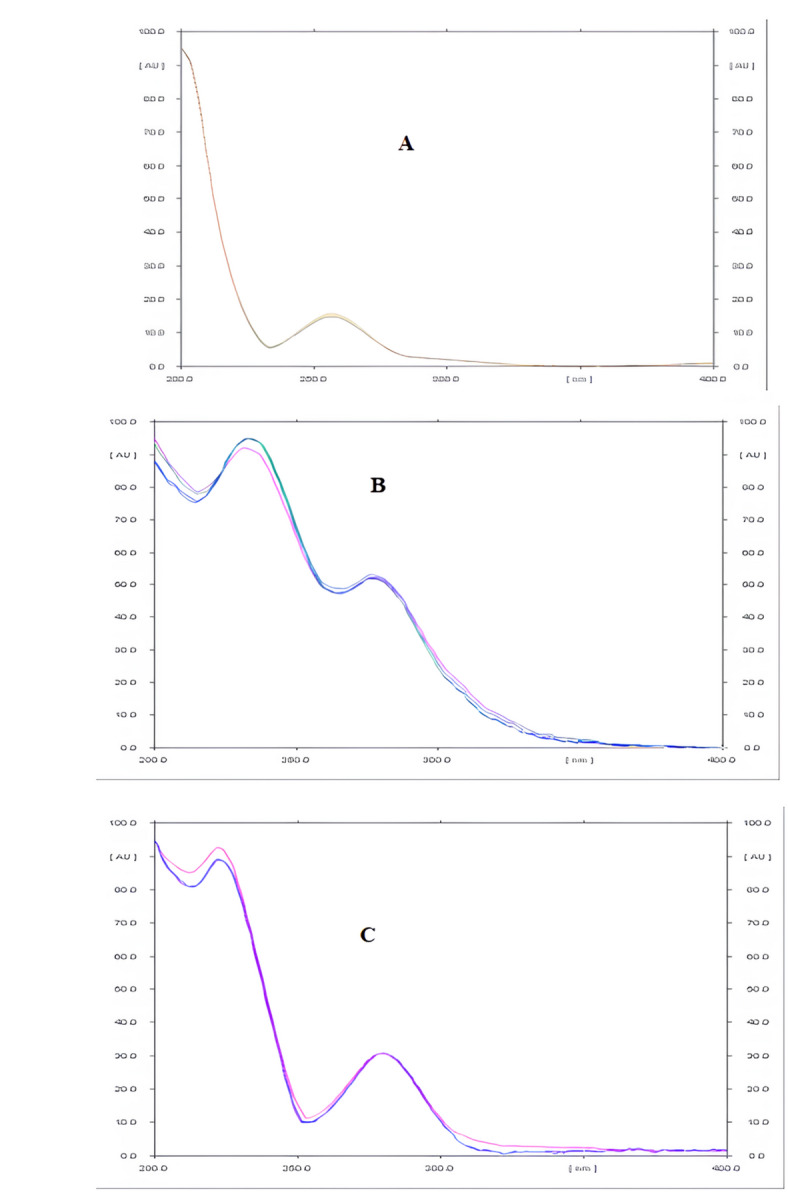
Superimposed UV spectra, obtained from the corresponding standard and extracted tablet solution utilizing the proposed HPTLC- densitometric method, illustrating peak purity of (**A**) PHE, (**B**) CPM, C) IBU.

The system suitability parameters were calculated, yielding satisfactory outcomes. Appropriate retardation factors (R_f_) were observed, with selectivity factors (α) of 5.08 and 2.19 for PHE, IBU, and CPM, respectively. The corresponding resolutions (R_s_) were 8.44 and 4.57 for PHE, IBU, and CPM, respectively. Tailing factors (T) were determined to be 1.0 ± 0.01, 1.0 ± 0.01, and 0.95 ± 0.01 for PHE, IBU, and CPM, respectively, as summarized in Table 1. Upon utilizing the optimized chromatographic conditions, efficient separation with sharp, and symmetrical peaks was attained for PHE, IBU, and CPM, with retardation factors of 0.25 ± 0.01, 0.63 ± 0.01, and 0.79 ± 0.01 for PHE, IBU, and CPM, respectively; Fig. 3a. Interday- variability of Rf, which checks variations in Rf within three different days, and Interplate - variability of Rf, which checks variations in Rf between different three plates on the same day were performed, as demonstrated in Table 1. Specificity was further affirmed utilizing the WinCATS spectral correlation tool through assessment of peak purity for each eluted drug; Fig. 4.

**Table 1:** System suitability parameters of the proposed HPTLC - densitometric and HPLC-DAD methods.

HPTLC - densitometric method				
Parameter	PHE	IBU	CPM	Reference value [38]
Retardation factor (R_f_) ^a^				
Mean ± SD ^b^	0.25 ± 0.01	0.63 ± 0.02	0.79 ± 0.02	
Mean ± SD ^c^	0.25 ± 0.01	0.59 ± 0.01	0.27 ± 0.01	
Capacity factor (k’) ^d^ (Mean ± SD)	3.00 ± 0.03	0.59 ± 0.02	0.27 ± 0.03	
Selectivity (α) ^e^	5.08	2.19		> 1
Resolution (Rs) ^f^	8.44	4.57		> 1.5
Tailing factor (T) (Mean ± SD)	1.00 ± 0.01	1.00 ± 0.01	0.95 ± 0.01	T ≤ 2

HPLC-DAD method				
Parameter	PHE	CPM	IBU	Reference value [38]
Retention time (T_R_) (min) (Mean ± SD)	2.97 ± 0.03	4.59 ± 0.02	5.46 ± 0.03	
Selectivity (α)^c^	1.90	1.36		> 1
Resolution (R_s_) ^d^	7.62	3.21		> 1.5
Tailing factor(T) (Mean ± SD)	1.12 ± 0.02	1.00 ± 0.01	0.96 ± 0.01	T ≤ 2
Column efficiency(N)	5122	5674	6256	*N* > 2000
Height equivalent to theoretical plates (HETP) (cm/plate)	4.88 × 10^− 3^	4.40 × 10^− 3^	3.99 × 10^− 3^	

^a^ Retardation factor (R_f_) = distance travelled by the analyte/distance travelled by the solvent front.

^b^ Interday- variability of R_f,_ which checks variations in R_f_ within three different days.

^C^ Interplate- variability of Rf, which checks variations in R_f_ between three different plates on the same day.

^d^ Capacity factor (k’) = (1 -R_f_) / R_f_.

^e^ Selectivity (α) = k’_2_/k’_1_ calculated for each of two successive peaks.

^f^ Resolution (R_s_) = Rf_2_ – Rf_1_ / 0.5 (w_1_ + w_2_), where R_f_ is the retardation factor, and w is the peak width calculated for each of two successive peaks for HPTLC and = 2(t_RB_- t_RA) / (_W_B+_W_A_) for HPLC.

Mean ± SD are average of three replicate injections.

#### Suitability parameters for HPLC-DAD

**Fig. 5:**
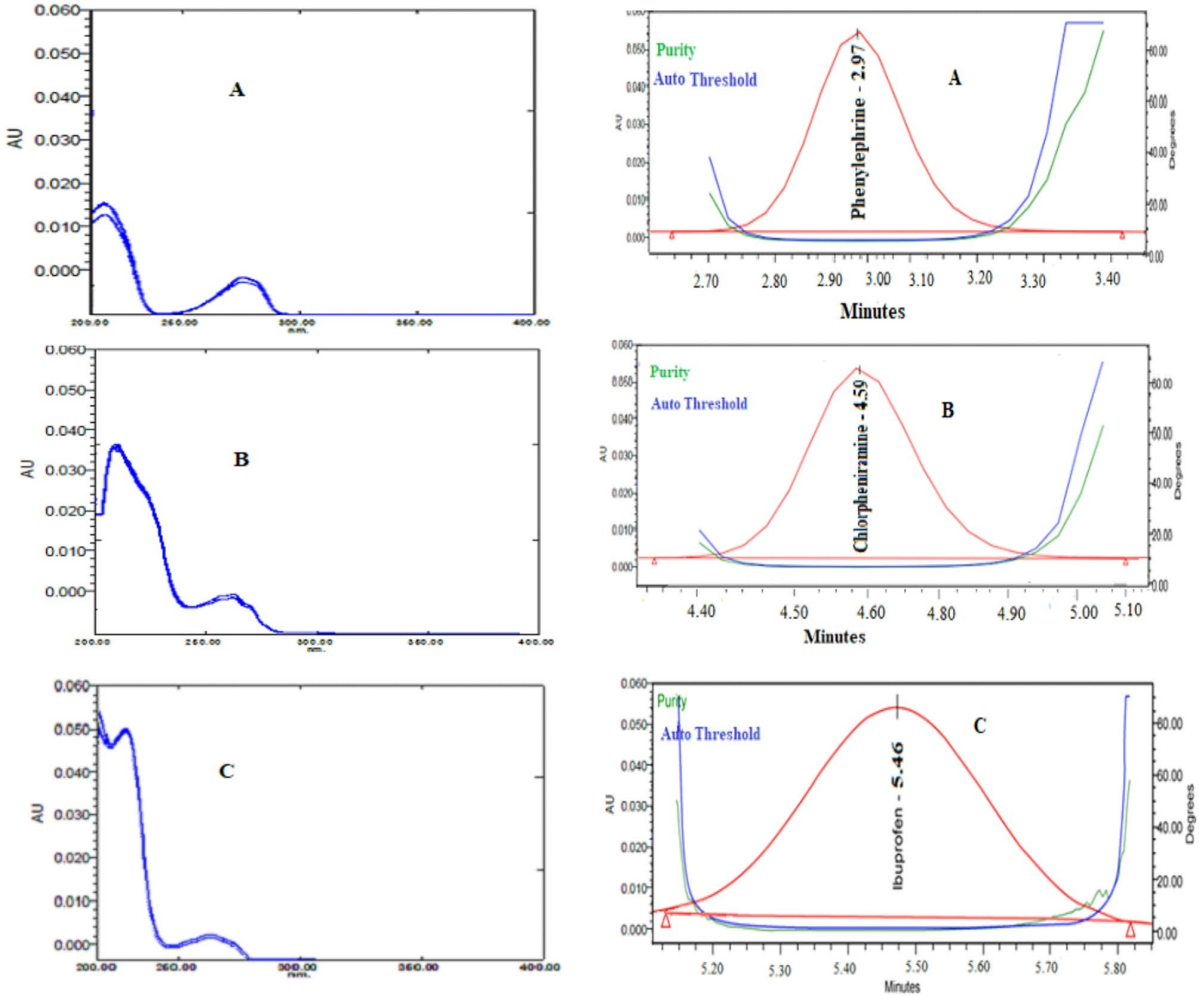
Superimposed UV spectra, and purity plots obtained from the corresponding standard and extracted tablet solution utilizing the proposed HPLC -DAD method, illustrating peak purity of (**A**) PHE, (**B**) CPM, and (**C**) IBU.

The parameters were computed and satisfactory findings were achieved with appropriate retention times (t_R_), selectivity factors (α) attained to be 1.90, and 1.36 for PHE, CPM, and IBU, respectively, with resolutions (Rs) of 7.62, and 3.21 for PHE, CPM, and IBU, respectively, whilst, tailing factors (T) were computed to be 1.12 ± 0.02, 1.00 ± 0.01, and 0.96 ± 0.01 for PHE, CPM, and IBU, respectively, with theoretical plate counts (N) determined to be 5122, 5674, and 6256 for PHE, CPM, and IBU, respectively, eventually heights equivalent to theoretical plate (HETP) were 4.88 × 10^− 3^, 4.40 × 10^− 3^,and 3.99 × 10^− 3^ cm/plate for PHE, CPM, and IBU, respectively as detailed in Table 1. Eventually, under the optimized chromatographic conditions, effective separation with sharp peaks with no tailing was achieved for PHE, CPM, and IBU, with retention times of 2.97 ± 0.03 min for PHE, 4.59 ± 0.02 min for CPM, and 5.46 ± 0.03 min for IBU, respectively while maleic acid peak was detected at about 2.1 min. The complete separation of all three analytes was accomplished within an approximate analysis time of 6.0 min; Fig. 3b. The specificity of detection was verified via utilizing a diode array detector by accurately identifying each component’s retention times and examining the corresponding UV spectra of the eluted components at their respective retention times, and assessment of peak purity for each eluted drug; Fig. 5.

### Method validation

The mentioned approaches were validated in accordance with ICH guidelines^40^, covering sensitivity, linearity, accuracy, precision. Linearity ranges were 0.1–14.0 µg/band, 0.1–10.0 µg/band and 2.0–40.0 µg/band in case of HPTLC and 0.2–24.0 µg/mL, 0.1–10.0 µg/mL, and 2.0–350.0 µg/mL in case of HPLC for PHE, CPM, and IBU, respectively IBU is present as the component of high concentration in the dosage form. Therefore, its calibration range should be extended to 350.0 µg/mL. This ensures compatibility with the other two co-formulated drugs, maintaining their linearity, accuracy, and precision within the specified range. The attained low % RSD values (≤ 2.0%), high recovery percentages, and stable retention times demonstrate that the method provides consistent and reproducible results without the need for an internal standard. LOD, LOQ which were experimentally verified by evaluating the signal-to-noise ratio of chromatographic peaks at low concentrations, where signal to noise ratios (S/N) of approximately 3:1 and 10:1 were obtained for LOD and LOQ, respectively, confirming adequate method sensitivity. The specificity of the methods was carefully evaluated with mixtures of the cited drugs with various ratios and good recoveries percentages and low SD. The recommended approaches’ specificity was ensured via analyzing the laboratory- prepared mixtures comprising PHE, CPM, and IBU in varying proportions, with sufficient findings attained within the defined calibration ranges, as illustrated in Tables 2 and S1.

**Table 2:** Regression and validation parameters of the proposed HPTLC-densitometric and HPLC-DAD methods.

Method parameter	HPTLC-densitometric method			HPLC- DAD method		
	PHE	CPM	IBU	PHE	CPM	IBU
Calibration range	0.1–14.0 µg/band	0.1–10.0 µg/band	2.0–40.0 µg/band	0.2–24.0 µg/mL	0.1–10.0 µg/mL	2.0-350.0 µg/mL
Regression equations parameters						
Slope (b) ^a^	763.81	737.26	748.88	27,744	48,692	6024
Intercept (a)	-399.09	489.62	-518.95	-3943	-4633	-1710
Correlation coefficient (r)	0.9998	0.9999	0.9999	0.9999	0.9999	0.9998
Accuracy (mean ± SD)	100.21 ± 0.98	99.62 ± 0.30	99.81 ± 0.40	99.99 ± 0.78	99.70 ± 0.70	100.15 ± 0.55
Precision						
% RSD ^b^	0.24	0.44	0.34	0.40	0.61	0.19
% RSD ^c^	0.42	0.75	0.76	0.42	0.69	0.23
SD of residuals	4.47	3.05	49.12	221.70	158.03	433.26
LOD ^d^	0.02	0.01	0.22	0.03	0.01	0.24
LOQ ^d^	0.06	0.03	0.66	0.09	0.03	0.73
Specificity (mean ± SD)	99.37 ± 0.65	99.94 ± 0.89	100.04 ± 0.48	100.04 ± 0.45	99.90 ± 0.39	100.04 ± 0.11
Robustness ^e^	0.87	0.52	0.49	0.32	0.38	0.47

^a^ Regression equation: *A* = *a* + *bc*, where ‘A’ is the average peak area and ‘c’ is the concentration.

^b^ Intra-day precision [average of three different concentration of three replicates each (*n* = 9) within the same day], for HPTLC, the concentrations were: (2.0, 6.0, 10.0 µg/band) for PHE (2.0, 4.0, 8.0 µg/band) for CPM, and (10.0, 20.0, 30.0 µg/band) for IBU. For HPLC: the concentrations were (6.0, 12.0, 18.0 µg/mL) for PHE, CPM (4.0, 6.0, 8.0 µg/mL), and (10.0, 150.0, 250.0 µg/mL) for IBU.

^c^ Inter-day precision [average of three different concentration of three replicates each repeated on three successive days], the concentrations were the same as in intra-day precision.

^d^ LOD and LOQ are calculated according to ICH, 3.3 × SD of the residuals/slope and 10 × SD of the residuals/slope, respectively.

^e^ For HPTLC: average of the change in scanning wavelength (± 1 nm), ethyl acetate ratio (± 1%) and saturation time (± 5 min). For HPLC: average for flow rate (± 0.1 mL/min), detection wavelength (± 1 nm), pH (± 0.1) temperature (± 2 º C).

Furthermore, the studied methodologies were applied to concurrently estimate PHE, CPM, and IBU in their pharmaceutical preparation, Advil allergy & sinus relief caplets. Ultimately, the resultant chromatograms showed that PHE, CPM, and IBU were well-resolved from any excipient peaks, with no interference observed at the retention times of the analytes. The approaches’ validity was additionally verified through the application of the standard addition technique, as detailed in Table 3. The standard addition technique is a powerful tool used in analytical methods to confirm the absence of matrix effects and excipient as well as accuracy of the proposed methods and efficiency of extraction steps. By spiking a known amount of analyte into the sample matrix and measuring the resulting response, any matrix effects or excipient interference can be accounted for and eliminated. This technique ensures that the observed signal is solely due to the analyte of interest, rather than any other compounds present in the sample. Through the use of standard addition, analysts can confidently determine the concentration of the analyte without worrying about the impact of the sample matrix. This approach provides a high degree of accuracy and reliability in analytical results. The accuracy of our chromatographic method was assessed by standard addition technique as mentioned in Table 3. Peak purity analysis confirmed the absence of co-eluting peaks as illustrated in Figs. 4 and 5. These results demonstrate that the method is selective and suitable for the intended pharmaceutical matrices, addressing potential matrix interferences effectively.

**Table 3:** Results obtained by applying the proposed HPTLC–densitometric and HPLC-DAD methods for the determination of PHE, CPM and IBUin Advil Allergy & Congestion relief caplets and application of standard addition technique.

Pharmaceutical formulation	HPTLC -densitometric method					HPLC -DAD method				
	Drug	% Found ± SD ^a^	Standard addition technique			Drug	% Found ± SD ^a^	Standard addition technique		
			Claimed ( µg/band)	Pure Added (µg/band)	% Recovery of the pure added ^b^			Claimed ( µg/mL)	Pure Added ( µg/mL)	% Recovery of the pure added ^b^
Advil Allergy & Congestion relief caplets (B. No. 070140 A; labeled to contain 10.0 mg PHE, 4.0 mg CPM, and 200 mg IBU, per caplet)	**PHE**	100.33 ±0.58	2.5	1.25	99.20	**PHE**	100.87 ± 0.31	5.0	2.5	99.60
				2.5	100.40				5.0	100.40
				5.0	99.40				10.0	99.30
			**Mean ± SD** 99.67 ± 0.64					**Mean ± SD** 99.77 ± 0.57		
	**CPM**	100.93 ± 0.98	1.0	0.5	100.60	**CPM**	99.70 ± 0.70	2.0	1.0	100.10
				1.0	100.30				2.0	99.50
				2.0	100.50				4.0	99.00
			**Mean ± SD** 100.47 ± 0.15					**Mean ± SD** 99.53 ± 0.55		
	**IBU**	99.18 ± 0.26	10.0	5 0.0	99.80	**IBU**	100.16 ± 0.62	100.0	50.0	100.36
				10.0	99.00				100.0	99.61
				20.0	99.60				200.0	99.86
			**Mean ± SD** 99.47 ± 0.42					**Mean ± SD** 99.94 ± 0.38		

^a^ Average of five determinations.

^b^ Average of three experiments.

Robustness was applied for the recovery percentages three different levels of certain selected parameters including those in the optimum conditions, scanning wavelength (± 1 nm), ethyl acetate ratio (± 1%) and saturation time (± 5 min) (for HPTLC), and in flow rate (± 0.1 mL/min), detection wavelength (± 1 nm), pH (± 0.1), and column temperature (± 2º C ) (for HPLC), as presented in Tables 2 and S5.

### Statistical analysis

A statistical evaluation of the results attained for PHE, CPM, and IBU in their pure forms utilizing the studied methodologies, compared with those achieved from the official methodologies^39^, demonstrated that the calculated *t* and *F* values were lower than their respective tabulated values at a significance level of *P* ≤ 0.05. This outcome affirms that, with respect to precision and accuracy, no critical variation was observed; Table S2. Additionally, a one-way ANOVA analysis was conducted to statistically evaluate the findings attained utilizing the studied methodology in comparison with those obtained through the official approaches^39^, and reported approach^36^. Ultimately, one-way ANOVA testing was performed to statistically assess the results attained from robustness studies in the proposed methods. This analysis demonstrated the absence of significant variation, as the calculated F value was lower than the critical value and the P value exceeded 0.05, the absence of statistically significant differences between the proposed, official^39^, and reported^36^ methods indicate analytical equivalence in terms of accuracy and precision, confirming the suitability of the developed HPLC-DAD method for routine quality control applications; Tables S3, S4 and S5. Moreover, statistical treatment of data was performed via calculating confidence intervals for slope and intercept for each calibration curve at confidence level of 95%, the resulting confidence intervals are consistently narrow, confirming calibration precision; Table S6. Also, both of relative and expanded uncertainty have been calculated and added in Table S6. For the HPTLC method, uncertainties remain consistently acceptable across all drugs (PHE = 1.2%, CPM = 0.80%, and IBU = 13%). Whilst for HPLC-DAD method, uncertainties were 1.6%, 0.65%, and 14.40% for PHE, CPM, and IBU, respectively. IBU still has higher uncertainty (14.40%) which is realistic and consistent with HPTLC (13%), but it referred to its larger residual scatter relative to its slope. Ultimately, both methods showed their high reliability for the target drugs.

### Applicability assessment

The assessment of the approaches’ applicability and functionality was accomplished via applying two advanced tools; CACI^41^, and BAGI^42^. The innovative CACI tool offers a straightforward, practical, effective, and comprehensible framework for the evaluation and comparison of analytical approaches, grounded in the essential principles of click analytical chemistry. Main parameters comprise sample size, sample preparation, sensitivity, applicability, feasibility, portability, and automation, each assigned a score to represent the method’s alignment with these concepts. The pictogram color coding in CACI indicates the technique’s performance with respect to each assessed parameter. Superior performance is represented by a colored pictogram, whereas gray indicates moderate practical performance, and black denotes insufficient performance or non-compliance with the defined parameters. The software is provided as an open-source platform, accessible at bit.ly/CACI2025. The BAGI tool is an innovative software developed to effectively evaluate the applicability of analytical methodologies. Serving as a complement to established green assessment matrices, it primarily emphasizes practical aspects. This tool evaluates ten main attributes including the type of analysis, the number of analytes that are simultaneously determined, the number of samples that can be analyzed per hour, the type of reagents and materials used in the analytical method, the required instrumentation, the number of samples that can be simultaneously treated, the requirement for pre-concentration, the automation degree, the type of sample preparation, and the amount of sample. A sequential blue color scale was employed to represent the eventual score, with dark blue, blue, light blue, and white indicating high, medium, low, and no compliance with the defined parameters, respectively. In terms of the total score attained, a value greater than 60 is recommended for an analytical approach to be considered practically applicable. The applicability of the analytical methodology is assessed via utilizing a dedicated web-based application (bagi-index.anvil.app). Evidently, CACI incorporates multiple factors that are not addressed within the BAGI software. These tools have certain limitations, as their scores can be influenced by subjective judgment in criteria weighting, the simplification of complex method characteristics into a single numerical value, and sensitivity to small changes in input data that can substantially impact the final score. They were employed as guidance for comparative purposes rather than as a definitive evaluation. The CACI and BAGI tools were employed to evaluate the official^39^ and reported HPLC^34–37^ methods, as presented in Tables 4, 5 and S7. Application of CACI tool to the proposed HPTLC and HPLC-DAD methods, achieved scores of 75, and 73, respectively, but the reported HPLC approaches^34–37^, and official method for PHE, CPM, and IBU yielded score values of 70, and 65, respectively. On the other hand, upon application of BAGI tool to the studied HPTLC and HPLC methods and the reported HPLC approaches^34–37^, they yielded a score value of 85, so they are more applicable than the official methods for PHE, CPM, and IBU that achieved a score value of 65. The studied approaches achieved higher CACI (75 and 73) and BAGI (85 and 85) scores for HPTLC, and HPLC-DAD, respectively, compared to the official^39^, and reported HPLC methods^34–37^, thereby confirming its strong applicability and functionality, due to their simplicity, cost-effectiveness, rapid separation, along with application of the proposed HPLC-DAD method in dissolution profiling studies.

**Table 4:** Applicability and greenness assessment of the proposed HPTLC-densitometric and HPLC-DAD, and reported HPLC methods using CACI, BAGI, AGSA, and CaFRI tools.

	CACI tool [41]	BAGI tool [42]	AGSA tool [50]	CaFRI tool [51]
Proposed HPTLC-densitometric method				
Proposed HPLC- DAD method				
Reported method [34]				
Reported method [35]				
Reported method [36]				
Reported method [37]				

**Table 5:** Greenness and Applicability assessment of the proposed HPLC-DAD, and reported HPLC methods using AGSA, and CaFRI, CACI, and BAGI tools, indicating direct quantitative comparison.

Method	Solvent consumption Total solvent volume = flow rate × total run time (V = F × t)	Run time	Greenness scores	Applicability scores
Proposed HPLC- DAD method	1.3 × 6 = 7.8 mL	6.0 min	AGSA: 84.72 CaFRI: 89.0	CACI: 73.0 BAGI: 85.0
Reported HPLC method [34]	1 × 19.5 = 19.5 mL	19.5 min	AGSA: 76.39 CaFRI: 85.0	CACI: 70.0 BAGI: 85.0
Reported HPLC method [35]	1.2 × 7 = 8.4 mL	7.0 min	AGSA: 76.39 CaFRI: 85.0	CACI: 70.0 BAGI: 85.0
Reported HPLC method [36]	0.25 × 6 = 1.5 mL	6.0 min	AGSA: 81.94 CaFRI: 85.0	CACI: 70.0 BAGI: 85.0
Reported HPLC method [37]	1 × 14 = 14 mL	14.0 min	AGSA: 76.39 CaFRI: 85.0	CACI: 70.0 BAGI: 85.0

### Greenness assessment

The analytical approaches’ greenness evaluation has become increasingly critical, with the primary aim of promoting ecological sustainability through the lessening or mitigation of hazardous solvents^1–3,43–49^. In green chromatographic practices, the total solvent consumption per run (V) is calculated as the product of flow rate and run time (V = F × t). In the proposed HPLC–DAD method, the total solvent volume consumed per run is 7.8 mL (1.3 mL min⁻¹ × 6 min). Accordingly, the developed method demonstrates improved environmental compatibility through its short run time, moderate flow rate, and moderate solvent consumption per run, in addition to the use of a relatively benign mobile phase composed of ethanol and ammonium acetate buffer, making the proposed method consistent with green analytical chemistry principles that emphasize reduced solvent usage and safer mobile phase composition. Therefore, advanced assessment tools, such as AGSA^50^, and CaFRI^51^, are required to evaluate the ecological impacts of the mentioned approaches. The AGSA tool, an Analytical Green Star Area software freely accessible at *bit.ly/AGSA2025*, employs an integrated and comprehensive scoring system to evaluate 12 concepts of Green Analytical Chemistry (GAC), illustrating the level of compliance through a star-shaped diagram. The AGSA tool assesses several parameters, including solvent type and consumption, reagent and chemical hazards, energy consumption, waste generation and disposal, occupational safety, sample preparation requirements, instrumentation and operational conditions, analysis time and throughput. Each concept is illustrated by a separate axis of the star, where the extent of compliance is indicated by the length of the respective axis. The advanced CaFRI tool constitutes an inclusive greenness assessment framework that identifies carbon footprint as the principal ecological impact parameter and is specifically designed for the assessment of the analytical laboratory practices. In the CaFRI assessment tool, the highest possible score achievable by any analytical approach is 100. The assessment considers multiple critical factors, including energy consumption, the carbon dioxide production, implementation of targeted carbon footprint reduction strategies, sample storage, transportation, personnel involvement, waste management, recycling initiatives to reduce resource consumption, and chemical usage. Energy usage in HPLC techniques is about 8–1.5 kWh per hour, depending on the detector and pump configuration. This tool is incorporated into user-oriented software platform, which is freely accessible online at (https://bit.ly/CaFRI). The footprint diagram is segmented into discrete regions corresponding to specific evaluative parameters, with red indicating suboptimal performance, yellow denoting moderate performance, and green representing ideal performance, in accordance with the principles of green chemistry. The values attained from the questionnaire are converted into a final score on a 0–100 scale, wherein a fully green procedure, as determined by the carbon footprint assessment, attains the maximum score of 100. The studied chromatographic approaches were compared to the official^39^ and reported HPLC approaches^34–37^, as illustrated in Tables 4 and S7. Additionally, a quantitative comparison was performed between the proposed HPLC-DAD method and reported HPLC approaches to prove superiority of the proposed HPLC - DAD method, including the main environmental and analytical parameters such as solvent consumption, total run time, and greenness and assessment scores; Table 5.Upon employing AGSA tool to the proposed HPTLC, and HPLC-DAD methods, as well as the reported method^36^, they yielded score values of 81.94, 84.72, and 81.94 respectively, whilst the reported HPLC approaches^34,35,37^, resulted in a score value of 76.39, and the official methods for PHE, CPM, and IBU achieved score values of 73.61, 73.61, and 70.83, respectively. On the other hand, CaFRI tool was applied to the studied HPTLC, and HPLC approaches, and achieved scores of 86, and 89, respectively, while the reported approaches^34–37^, and official methods for PHE, CPM, and IBU, yielded score values of 85, and 80, respectively. Therefore, the proposed chromatographic approaches demonstrated superior performance over the reported HPLC methods and official approaches. This enhanced performance is ascribed to lower instrumental energy consumption, reduced solvent usage through the employment of environmentally benign solvents such as ethanol, and notable efforts in minimizing the carbon footprint, as reflected by elevated greenness scores for AGSA (81.94 and 84.72), and CaFRI (86 and 89) for HPTLC, and HPLC, respectively.

### In-vitro dissolution monitoring of Advil allergy and sinus relief caplets

**Fig. 6:**
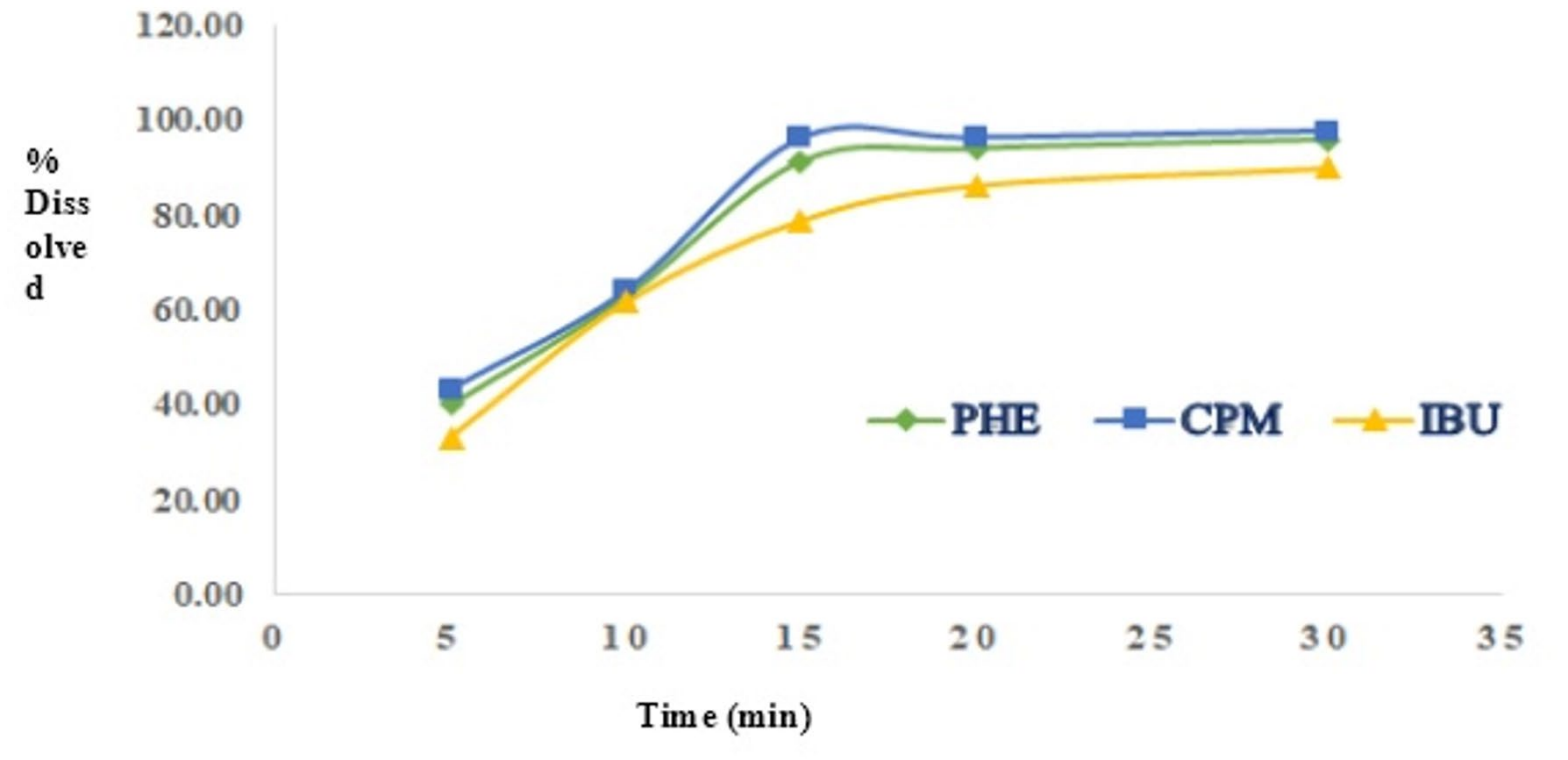
Dissolution profiles of PHE, CPM, and IBU from Advil allergy & sinus relief caplets in 50 mM potassium phosphate buffer with pH 6.5 as a dissolution medium.

In vitro dissolution monitoring constitutes a fundamental step in drug manufacturing and development, serving to elucidate the relationship between in vitro and in vivo profiles and to assess the quality of pharmaceutical formulations^16,38^. In our study, which focused on dosage forms and in vitro dissolution testing, the use of an internal standard was not deemed necessary. This is because in such controlled environments, the extraction procedures are straightforward, and the matrix effects are minimal or consistent, reducing the potential of variability caused by endogenous substances. Since in vitro dissolution studies involve relatively simple sample matrices and standardized procedures, the potential for matrix effects that could enhance or suppress the analyte signal is significantly ignored. Consequently, the internal standard technique, which is primarily crucial for compensating for variability in complex biological matrices like plasma, can be effectively omitted without compromising the accuracy or reliability of our results as provided in Table 6. Furthermore, chromatographic selectivity was carefully assessed throughout all dissolution sampling intervals to ensure reliable quantification of the analyte. The proposed HPLC-DAD methodology exhibited constant selectivity across all time intervals, with no significant alterations in retention times, or peaks resolution and shapes during the dissolution profiling. Furthermore, potential interference from formulation excipients was systemically evaluated via analyzing dissolution samples collected at various time intervals. The results affirmed the absence of co-eluting peaks at the retention times of the target analytes, demonstrating that excipients didn’t interfere with the chromatographic determination. Dissolution monitoring of Advil allergy & sinus relief caplets was conducted following USP recommendations^38^, utilizing a dissolution medium of 50 mM potassium phosphate buffer with pH 6.5 as the dissolution medium. In the present study, the release percentage of the specified analytes from Advil allergy & sinus relief caplets in 50 mM potassium phosphate buffer with pH 6.5 surpassed 90% within 30 min for PHE, CPM, and IBU. The attained results demonstrated that the studied formulation provided adequate and reproducible drug release behavior comparable to the expected drug release profiles for the target analytes in accordance with USP recommendations^38^, and within the acceptable regulatory criteria. For PHE, USP generally expects not less than 75% (Q) drug release within 45 min, whilst, CPM, the USP monograph specifies that not less than 80% (Q) of the labeled amount should be dissolved within 30 min., and IBU, pharmacopeial and regulatory guidance typically requires around 80% or more drug release within about 60 min. Thus, the established acceptance criteria, defined by the quantity (Q) of active ingredient released within a specified time frame, are fulfilled. Dissolution profiles were generated via plotting the percentage of dissolved drug as a function of time, as depicted in Fig. 6. Furthermore, the variability in dissolution data was expressed as mean values and standard deviations of multiple determinations at each time interval. The variability of the dissolution data was minimal and within acceptable limits (SD ≤ 2), indicating good reproducibility and reliability of the results, as demonstrated in Table 6.

**Table 6:** Dissolution profiles for PHE, CPM, and IBU, along with variability in dissolution data.

Time (min)	PHE (Mean %released ± SD) *n* = 6	CPM (Mean %released ± SD) *n* = 6	IBU (Mean %released ± SD) *n* = 6
5	40.18 ± 1.12	43.2 ± 0.62	33.02 ± 0.23
10	62.98 ± 0.96	63.95 ± 0.58	61.63 ± 1.14
15	91.38 ± 0.72	96.17 ± 0.91	78.81 ± 0.78
20	94.12 ± 0.85	96.32 ± 1.05	86.2 ± 0.68
30	96.03 ± 0.92	97.71 ± 0.36	91.12 ± 0.98

## Conclusion

Two eco-friendly chromatographic methods, HPTLC densitometry and HPLC-DAD, were successfully developed and validated for the simultaneous determination of phenylephrine (PHE), chlorpheniramine (CPM), and ibuprofen (IBU) in bulk forms, laboratory prepared mixtures, and a commercial cold/flu caplet formulation, with the HPLC-DAD method further applied to in‑vitro dissolution profiling. Both methods demonstrated excellent accuracy, precision (RSD ≤ 2.0%), sensitivity, and selectivity, with the HPLC-DAD method offering a notably shorter analysis time (less than 6 min) compared to the previously reported methods, thereby supporting high‑throughput quality control operations. The greenness and practical applicability of the proposed approaches were rigorously evaluated using four contemporary metric tools; CACI, BAGI, AGSA, and CaFRI, yielding scores that were comparable to or higher than those of official pharmacopeial methods and previously reported HPLC procedures, underscoring their alignment with current principles of sustainable analytical chemistry. The proposed methods represent a practical, environmentally conscious, and regulatory ready alternative for the routine quality control of this ternary combination.

## Supplementary Information

Supplementary Material 1

## Data Availability

All data generated or analysed during this study are included in this published article.

## References

[1] Gałuszka, A., Migaszewski, Z. & Namieśnik, J. The 12 principles of green analytical chemistry and the SIGNIFICANCE mnemonic of green analytical practices. *TRAC Trends Anal. Chem.* **50**, 78–84 (2013).

[2] Kurowska-Susdorf, A. et al. Green analytical chemistry: Social dimension and teaching. *TRAC Trends Anal. Chem.* **111**, 185–196 (2019).

[3] Nabil, M., Ahmed, D. A., Abbas, S. S., Lotfy, H. M. & Marzouk, H. M. Green HPLC strategy for quantification of carvedilol and hydrochlorothiazide in cardiac medications with in-vitro dissolution kinetics and impurity profiling. *BMC Chem.* **19** (1), 1–13 (2025). 40611289 10.1186/s13065-025-01559-2 PMC12224793

[4] Darweish, E., Eissa, M. S., Fayez, Y. M. & Marzouk, H. M. Chromatographic estimation of a novel triple-therapy combination targeting Helicobacter pylori eradication in different matrices. *Bioanalysis* **13** (20), 1547–1557 (2021). 34708661 10.4155/bio-2021-0183

[5] Marzouk, H. M., Ibrahim, E. A., Hegazy, M. A. & Saad, S. S. Greenness profile assessment of selective liquid chromatographic methods for determination of a quaternary antimigraine combination along with three of their related official impurities. *Biomed. Chromatogr.* **35** (9), e5132 (2021). 33792069 10.1002/bmc.5132

[6] Płotka, J. et al. Green chromatography. *J. Chromatogr. A*. **1307**, 1–20 (2013). 23932374 10.1016/j.chroma.2013.07.099

[7] Coskun, O., Akbarzadeh, S. & Günçer, B. Different chromatographic techniques and recent advancements for biomedical and pharmaceutical applications. *North. Clin. Istanbul*. **12** (6), 739 (2025). 10.14744/nci.2025.42966 PMC12821153 41574145

[8] Parys, W., Dołowy, M. & Pyka-Pająk, A. Significance of chromatographic techniques in pharmaceutical analysis. *Processes* **10** (1), 172 (2022).

[9] Kelani, K. M., Fayez, Y. M., Abdel-Raoof, A. M., Fekry, R. A. & Hassan, S. A. Development of an eco-friendly HPLC method for the stability indicating assay of binary mixture of ibuprofen and phenylephrine. *BMC Chem.* **17** (1), 141 (2023). 37876006 10.1186/s13065-023-01056-4 PMC10598928

[10] Fuchs, B., Süß, R., Teuber, K., Eibisch, M. & Schiller, J. Lipid analysis by thin-layer chromatography—a review of the current state. *J. Chromatogr. A*. **1218** (19), 2754–2774 (2011). 21167493 10.1016/j.chroma.2010.11.066

[11] Poole, C. F. Thin-layer chromatography: challenges and opportunities. *J. Chromatogr. A*. **1000** (1–2), 963–984 (2003). 12877208 10.1016/s0021-9673(03)00435-7

[12] Poole, C. F. & Poole, S. K. Progress in densitometry for quantitation in planar chromatography. *J. Chromatogr. B Biomed. Sci. Appl.* **492**, 539–584 (1989). 10.1016/s0378-4347(00)84479-5 2671002

[13] El-Gizawy, S. M., Atia, N. N., Ali, M. F. & Rushdy, D. H. Development of a highly sensitive and eco-friendly high-performance thin-layer chromatography approach for the determination of empagliflozin, pioglitazone, and rosuvastatin simultaneously in pharmaceutical preparations and different biological fluids. *JPC–Journal Planar Chromatography–Modern TLC*. **36** (5), 401–414 (2023).

[14] Do, T. K. T. & Reich, E. Insights into the evolution and future of high-performance thin-layer chromatography in routine quality control: A review. *JPC–Journal Planar Chromatography–Modern TLC*. **36** (5), 317–325 (2023).

[15] AlSalem, H. S. et al. High performance thin layer chromatography (HPTLC) analysis of anti-asthmatic combination therapy in pharmaceutical formulation: assessment of the method’s greenness and blueness. *Pharmaceuticals* **17** (8), 1002 (2024). 39204107 10.3390/ph17081002 PMC11357029

[16] Dressman, J. B. & Krämer, J. *Pharmaceutical dissolution testing* (Taylor & Francis Boca Raton, FL, 2005).

[17] Cardot, J., Beyssac, E. & Alric, M. In vitro-in vivo correlation: importance of dissolution in IVIVC. *Dissolution Technol.* **14** (1), 15 (2007).

[18] Anand, O., Yu, L. X., Conner, D. P. & Davit, B. M. Dissolution testing for generic drugs: an FDA perspective. *AAPS J.* **13**, 328–335 (2011). 21479700 10.1208/s12248-011-9272-y PMC3160163

[19] Ranjan, A., Zugah, M. N., Verma, R. K., De Beer, T. & Kumar, A. Mechanistic understanding of drug release in dissolution apparatuses–In-depth review. *J. Controlled Release.* 114754. (2026). 10.1016/j.jconrel.2026.114754 41759786

[20] Lutfor, M., Al, M., Nadarkhani, F. & Nezab, M. Advanced Dissolution Testing for Novel Drug Formulations: Challenges, Emerging Methods, and Regulatory Perspectives. *J. Angiotherapy*. **9** (1), 1–16 (2025).

[21] Marzouk, H. M., Rezk, M. R., Gouda, A. S. & Abdel-Megied, A. M. A novel stability-indicating HPLC-DAD method for determination of favipiravir, a potential antiviral drug for COVID-19 treatment; application to degradation kinetic studies and in-vitro dissolution profiling. *Microchem. J.* **172**, 106917 (2022). 34667334 10.1016/j.microc.2021.106917 PMC8518200

[22] Wadie, M., Abdel-Moety, E. M., Rezk, M. R. & Marzouk, H. M. A green and versatile HPLC-DAD method for determination of silodosin and solifenacin in their newly combined formulation: Evaluation of content uniformity and dissolution profiles. *Sustainable Chem. Pharm.* **44**, 101923 (2025).

[23] Wadie, M., Abdel-Moety, E. M., Rezk, M. R. & Marzouk, H. M. A novel eco-friendly HPLC method with dual detection modes for versatile quantification of dutasteride and silodosin in pharmaceutical formulation, dissolution testing and spiked human plasma. *Microchem. J.* **197**, 109753 (2024).

[24] Manukonda, V., Dandamudi, S. P., Kusuma, P. K., Thumma, G. & Gangarapu, K. Development and validation of a UPLC-MS/MS method for the simultaneous estimation of ertugliflozin and sitagliptin in bulk and tablet dosage forms: assessment of greenness and blueness. *Green. Anal. Chem.* **12**, 100193 (2025).

[25] Palermiti, A. et al. UHPLC-MS/MS method for the simultaneous quantification of five calcium channel antagonists’ drugs in human plasma. *Biomed. Pharmacotherapy*. **184**, 117873 (2025). 10.1016/j.biopha.2025.117873 39913972

[26] Sánchez-Sánchez, E. et al. Consumption of over-the-counter drugs: prevalence and type of drugs. *Int. J. Environ. Res. Public Health*. **18** (11), 5530 (2021). 34064096 10.3390/ijerph18115530 PMC8196755

[27] Eccles, R. Efficacy and safety of over-the‐counter analgesics in the treatment of common cold and flu. *J. Clin. Pharm. Ther.* **31** (4), 309–319 (2006). 16882099 10.1111/j.1365-2710.2006.00754.x

[28] Desjardins, P. J. & Berlin, R. G. Efficacy of phenylephrine. *Br. J. Clin. Pharmacol.* **64** (4), 555 (2007). 17610531 10.1111/j.1365-2125.2007.02935.x PMC2048561

[29] Yehia, A. M., Nabil, M., Badawey, A. M. & Abbas, S. S. Spectral resolution of quaternary components in a sinus and congestion mixture; Multivariate algorithms to approach extremes of concentration levels. *Spectrochim. Acta Part A Mol. Biomol. Spectrosc.* **239**, 118489 (2020). 10.1016/j.saa.2020.118489 32473561

[30] Najjar, T., Al-Alsheikh, O., Al-Dhawailie, A. & Shereif, A. Bioequivalence and pharmacokinetics of chlorpheneramine in healthy human volunteers. *Int. J. Clin. Pharmacol. Ther.* **33** (11), 619–622 (1995). 8688987

[31] Rainsford, K. Ibuprofen: pharmacology, efficacy and safety. *Inflammopharmacology* **17** (6), 275–342 (2009). 19949916 10.1007/s10787-009-0016-x

[32] Eccles, R., Fietze, I. & Rose, U-B. Rationale for treatment of common cold and flu with multi-ingredient combination products for multi-symptom relief in adults. *Open. J. Respiratory Dis.* **4** (3), 73–82 (2014).

[33] Farrer, F. Combination cold and flu preparations: what the pharmacist’s assistant should know: colds and flu. *SA Pharmacist’s Assistant*. **12** (1), 22–26 (2012).

[34] Baviskar, V., Donda, S., Patil, S., Deshmukh, P. & Patil, P. Article Details Development and validation of determination for chlorpheniramine maleate, phenylephrine hydrochloride and ibuprofen in bulk and pharmaceutical formulation by RP-HPLC method. *Indian Drugs* (2015).

[35] Abu El-Enin, M. A., Salem, Y. A., El‐Ashry, S. M. & Hammouda, M. E. Applying eco‐friendly micellar liquid chromatography for the simultaneous determination of two ternary mixtures utilized for cold treatment using monolithic column. *J. Chin. Chem. Soc.* **68** (9), 1686–1696 (2021).

[36] Ibrahim, M. M. & Bassuoni, Y. F. A computational optimized and validated eco-friendly and sustainable UHPLC method of a combined ternary mixture of chlorpheniramine maleate, ibuprofen and phenylephrine HCl: Application in their pharmaceutical formulation. *Microchem. J.* **212**, 113312 (2025).

[37] Sanchaniya, P. M., Mehta, F. A. & Uchadadiya, N. B. Development and Validation of an RP-HPLC Method for Estimation of Chlorpheniramine Maleate, Ibuprofen, and Phenylephrine Hydrochloride in Combined Pharmaceutical Dosage Form. *Chromatogr. Res. Int.* **2013** (1), 424865 (2013).

[38] The United States Pharmacopeia and The National Formulary. *USP 42-NF 37* (U.S, Pharmacopeial Convention., 2019).

[39] British Pharmacopeia vII. British Pharmacopeia Commission. London, TSO, UK. (2022).

[40] Guideline, I. *Validation of analytical procedures Q2 (R2)* (Geneva, Switzerland., 2022).

[41] Mansour, F. R., Bedair, A. & Locatelli, M. Click Analytical Chemistry Index as a novel concept and framework, supported with open source software to assess analytical methods. *Adv. Sample Preparation*. **14**, 100164 (2025).

[42] Manousi, N., Wojnowski, W., Płotka-Wasylka, J. & Samanidou, V. Blue applicability grade index (BAGI) and software: a new tool for the evaluation of method practicality. *Green Chem.* **25** (19), 7598–7604 (2023).

[43] Michael, A. M., Lotfy, H. M. & Nessim, C. K. Greenness profile and whiteness assessment of the stability-indicating HPLC method for the assay of levetiracetam. *Microchem. J.* **190**, 108669 (2023).

[44] Rostom, Y., Wadie, M., Rezk, M. R., Marzouk, H. M. & Abdel-Moety, E. M. Fingerprinting and iso-absorptive resolution techniques for the spectrally overlapping Dutasteride and Silodosin mixture: Content uniformity testing along with greenness profile assessment. *Spectrochim. Acta Part A Mol. Biomol. Spectrosc.* **273**, 121063 (2022). 10.1016/j.saa.2022.121063 35219273

[45] Nabil, M., Marzouk, H. M., Ahmed, D. A., Abbas, S. S. & Lotfy, H. M. Risk assessment based on spectrophotometric signals used in eco-friendly analytical scenarios for estimation of carvedilol and hydrochlorothiazide in pharmaceutical formulation. *Sci. Rep.* **14** (1), 19657 (2024). 39179633 10.1038/s41598-024-69746-0 PMC11343851

[46] Akabari, A. H. et al. White analytical chemistry–aligned eco-friendly, stability-indicating HPLC and HPTLC methods for simultaneous estimation of efonidipine hydrochloride ethanolate and chlorthalidone. *J. Taibah Univ. Sci.* **20** (1), 2606450 (2026).

[47] Shah, M., Patel, H. U. & Akabari, A. H. Eco-friendly HPTLC method for simultaneous estimation of gallic acid, ellagic acid, and curcumin biomarker in herbal formulation. *Essent. Chem.* **1** (1), 1–10 (2024).

[48] Rabadiya, V. A., Shah, N. & Akabari, A. H. Eco-friendly and stability-indicating HPTLC method for the estimation of Carvedilol in pharmaceutical dosage forms: a greenness assessment using NEMI scale, AGREE, and white analytical chemistry. *Green. Anal. Chem.* **13**, 100237 (2025).

[49] Rabadiya, V. A., Shah, N. & Akabari, A. H. Eco-friendly RP‐HPLC method for simultaneous estimation of amlodipine besylate and indapamide: analytical quality by design approach and greenness assessment. *Sep. Sci. Plus*. **8** (3), e70019 (2025).

[50] Mansour, F. R., Bedair, A., Belal, F., Magdy, G. & Locatelli, M. Analytical Green Star Area (AGSA) as a new tool to assess greenness of analytical methods. *Sustainable Chem. Pharm.* **46**, 102051 (2025).

[51] Mansour, F. R. & Nowak, P. M. Introducing the carbon footprint reduction index (CaFRI) as a software-supported tool for greener laboratories in chemical analysis. *BMC Chem.* **19** (1), 1–11 (2025). 40346688 10.1186/s13065-025-01486-2 PMC12065229

