# Supplementary material for: Green and applicable chromatographic approaches for the estimation of a multi-component cold and flu relief formulation along with in-vitro dissolution profiling
Source: Sci Rep. 2026 Jun 10;16:18026. doi: 10.1038/s41598-026-55497-7 (PMC13254206; doi:10.1038/s41598-026-55497-7)

| HPTLC - densitometric method | | | | | | | | | |
| --- | --- | --- | --- | --- | --- | --- | --- | --- | --- |
|  | **Claimed Conc. (μg/band)** | | | **Found Conc. (μg/band)** | | | **Recovery, %^a^** | | |
| Ratio PHE: CPM: IBU | **PHE** | **CPM** | **IBU** | **PHE** | **CPM** | **IBU** | **PHE** | **CPM** | **IBU** |
| 2.5: 1: 50 ^b^ | 1.00 ^b^ | 0.40 ^b^ | 20.00 ^b^ | 0.99 | 0.40 | 19.98 | 99.00 | 100.25 | 99.90 |
| 1: 1: 15 | 2.00 | 2.00 | 30.00 | 1.98 | 1.98 | 29.97 | 99.00 | 99.00 | 99.90 |
| 2: 1: 7.5 | 8.00 | 4.00 | 30.00 | 7.95 | 4.03 | 30.12 | 99.38 | 100.75 | 100.40 |
| 5: 1: 50 | 2.50 | 0.50 | 25.00 | 2.48 | 0.50 | 25.24 | 99.20 | 100.80 | 100.96 |
| 1: 1: 6 | 5.00 | 5.00 | 30.00 | 4.96 | 4.95 | 29.87 | 99.20 | 99.00 | 99.57 |
| 2.5: 2: 50 | 1.25 | 1.00 | 25.00 | 1.26 | 0.99 | 24.96 | 100.80 | 99.00 | 99.84 |
| 2.5: 1: 75 | 1.00 | 0.40 | 30.00 | 0.99 | 0.40 | 29.92 | 99.00 | 100.75 | 99.73 |
| Mean |  |  |  |  |  |  | **99.37** | **99.94** | **100.04** |
| SD |  |  |  |  |  |  | **0.65** | **0.89** | **0.48** |
| HPLC - DAD method | | | | | | | | | |
|  | **Claimed Conc. (μg/mL)** | | | **Found Conc. (μg/mL)** | | | **Recovery, %^a^** | | |
| Ratio PHE: CPM: IBU | **PHE** | **CPM** | **IBU** | **PHE** | **CPM** | **IBU** | **PHE** | **CPM** | **IBU** |
| 2.5: 1: 50 ^b^ | 5.00 ^b^ | 2.00 ^b^ | 100.00 ^b^ | 4.98 | 2.01 | 99.98 | 99.60 | 100.50 | 99.98 |
| 1: 1: 20 | 0.50 | 0.50 | 10.00 | 0.50 | 0.50 | 9.99 | 100.80 | 99.60 | 99.90 |
| 1.5: 1: 12.5 | 6.00 | 4.00 | 50.00 | 5.99 | 3.98 | 49.99 | 99.83 | 99.50 | 99.98 |
| 2: 1: 25 | 12.00 | 6.00 | 150.00 | 11.98 | 5.99 | 150.21 | 99.83 | 99.83 | 100.14 |
| 2: 1: 40 | 10.00 | 5.00 | 200.00 | 9.98 | 5.02 | 200.31 | 99.80 | 100.40 | 100.16 |
| 4.5: 2: 63 | 18.00 | 8.00 | 250.00 | 17.98 | 7.98 | 249.95 | 99.89 | 99.75 | 99.98 |
| 5: 1: 75 | 20.00 | 4.00 | 300.00 | 20.11 | 3.99 | 300.52 | 100.55 | 99.75 | 100.17 |
| Mean |  |  |  |  |  |  | **100.04** | **99.90** | **100.04** |
| SD |  |  |  |  |  |  | **0.45** | **0.39** | **0.11** |

**Table S1:** Determination of PHE, CPM, and IBU in laboratory prepared mixtures by the proposed HPTLC- densitometric and HPLC-DAD methods.

^a^  Average of three determinations.

^b^ Ratio of PHE, CPM, and IBU in the pharmaceutical formulation.

**Table S2:** Statistical comparison between the results obtained by the proposed HPTLC and HPLC-DAD method and official methods B.P. [39] for the determination of the proposed mixture in their pure powdered form.

| Items | HPTLC – densitometric method | | | | | | HPLC- DAD method | | | | | |
| --- | --- | --- | --- | --- | --- | --- | --- | --- | --- | --- | --- | --- |
|  | **PHE** | | **CPM** | | **IBU** | | **PHE** | | **CPM** | | **IBU** | |
|  | **The proposed HPTLC method** | **Official method ^a^** | **The proposed HPTLC method** | **Official method ^b^** | **The proposed HPTLC method** | **Official method ^c^** | **The proposed HPLC- DAD method** | **Official method ^a^** | **The proposed HPLC- DAD method** | **Official method ^b^** | **The proposed HPLC- DAD method** | **Official method ^c^** |
| Mean | 99.81 | 100.24 | 99.85 | 100.10 | 100.19 | 100.01 | 100.52 | 100.24 | 100.05 | 100.10 | 100.28 | 100.01 |
| SD | 0.58 | 0.70 | 0.84 | 0.64 | 0.60 | 0.51 | 0.82 | 0.70 | 0.80 | 0.64 | 0.41 | 0.51 |
| n | 6 | 5 | 7 | 5 | 6 | 5 | 6 | 5 | 6 | 5 | 6 | 5 |
| Variance | 0.336 | 0.490 | 0.706 | 0.410 | 0.360 | 0.260 | 0.672 | 0.490 | 0.640 | 0.410 | 0.168 | 0.260 |
| Student's-t test ^d^ (2.262) | 1.095 | ------- | 0.584 | ------- | 0.538 | ------- | 0.611 | ------- | 0.115 | ------- | 0.954 | ------- |
| F-test | 1.456 (5.19) | ------- | 1.722 (5.29) | ------- | 1.384 (5.394) | ------- | 1.372 (5.394) | ------- | 1.562 (5.394) | ------- | 1.547 (5.19) | ------- |

^a^ For Phenylephrine: Potentiometric titration method using ethanolic sodium hydroxide as a titrant.

^b^ For Chlorpheniramine: Potentiometric titration method using 0.1 M perchloric acid as a titrant.

^c^ For Ibuprofen: Titrimetric method using 0.1 M sodium hydroxide as a titrant.

^d^ Student’s t test tabulated can be calculated using two-tails at confidence level 95%.

**Table S3**: One way ANOVA testing for the proposed and official methods B.P. [39] used for the determination of the cited mixture.

|  | Source of Variation | DF | Sum of squares | Mean Square | F value | P- value |
| --- | --- | --- | --- | --- | --- | --- |
| PHE | **Between Groups** | 2 | 1.521 | 0.760 | 1.423 (3.738) | 0.273 |
|  | **Within Groups** | 14 | 7.482 | 0.534 |  |  |
| CPM | **Between Groups** | 2 | 0.213 | 0.106 | 0.169 (3.682) | 0.845 |
|  | **Within Groups** | 15 | 9.434 | 0.628 |  |  |
| IBU | **Between Groups** | 2 | 0.191 | 0.095 | 0.334 (3.738) | 0.720 |
|  | **Within Groups** | 14 | 3.995 | 0.285 |  |  |

The values between parentheses are the theoretical F values.

The population means are not significantly different.

**Table S4**: One way ANOVA testing for the proposed HPTLC–densitometric, HPLC-DAD and reported UHPLC methods [36] used for the determination of the cited drugs in their pure forms.

|  | Source of Variation | DF | Sum of squares | Mean Square | F value | P- value |
| --- | --- | --- | --- | --- | --- | --- |
| PHE | **Between Groups** | 2 | 1.651 | 0.825 | 1.90 (3.682) | 0.184 |
|  | **Within Groups** | 15 | 6.525 | 0.435 |  |  |
| CPM | **Between Groups** | 2 | 1.117 | 0.558 | 0.715 (3.634) | 0.504 |
|  | **Within Groups** | 16 | 12.496 | 0.781 |  |  |
| IBU | **Between Groups** | 2 | 0.341 | 0.170 | 0.668 (3.682) | 0.527 |
|  | **Within Groups** | 15 | 3.823 | 0.255 |  |  |

The values between parentheses are the theoretical F values.

The population means are not significantly different.

Reported UHPLC utilizes C_8_ column, (2.1 × 150, 1.7 μm), methanol: water: triethylamine (95:5:0.1, by volume) as mobile phase, pH was adjusted at 3 using *o*-phosphoric acid, and UV detection 220 nm.

**Table S5**: One way ANOVA testing for results attained by robustness studies performed in the proposed methods used for the determination of the cited drugs in their pure forms.

| HPTLC – densitometric method | | | | | | |
| --- | --- | --- | --- | --- | --- | --- |
|  | **Source of Variation** | **DF** | **Sum of squares** | **Mean Square** | **F value** | **P- value** |
| PHE | **Between Groups** | 2 | 0.079 | 0.040 | 1.030 (5.143) | 0.413 |
|  | **Within Groups** | 6 | 0.2304 | 0.038 |  |  |
| CPM | **Between Groups** | 2 | 0.355 | 0.178 | 0.452 (5.143) | 0.657 |
|  | **Within Groups** | 6 | 2.361 | 0.394 |  |  |
| IBU | **Between Groups** | 2 | 0.449 | 0.224 | 0.806 (5.143) | 0.489 |
|  | **Within Groups** | 6 | 1.671 | 0.278 |  |  |
| HPLC – DAD method | | | | | | |
| PHE | **Between Groups** | 3 | 0.206 | 0.069 | 2.352 (4.066) | 0.148 |
|  | **Within Groups** | 8 | 0.233 | 0.030 |  |  |
| CPM | **Between Groups** | 3 | 0.798 | 0.267 | 0.893 (4.066) | 0.485 |
|  | **Within Groups** | 8 | 2.382 | 0.298 |  |  |
| IBU | **Between Groups** | 3 | 0.618 | 0.206 | 0.840 (4.066) | 0.509 |
|  | **Within Groups** | 8 | 1.963 | 0.245 |  |  |

This study was applied for three different levels including the optimum conditions with slight variations in scanning wavelength (± 1 nm), ethyl acetate ratio (±1 %) and saturation time (± 5 min) (for HPTLC), and in flow rate (±0.1 mL/min), detection wavelength (±1 nm), pH (±0.1), and column temperature (± 2º C) (for HPLC).

The values between parentheses are the theoretical F values.

The population means are not significantly different.

| **Method Parameter** | **HPTLC-densitometric method** | | | **HPLC- DAD method** | | |
| --- | --- | --- | --- | --- | --- | --- |
|  | **PHE** | **CPM** | **IBU** | **PHE** | **CPM** | **IBU** |
| **n** | 6 | 7 | 6 | 6 | 6 | 6 |
| **Slope (b) ^a^** | 763.81 | 737.26 | 748.88 | 27744 | 48692 | 6024 |
| **Intercept (a)** | -399.09 | 489.62 | -518.95 | -3943 | -4633 | -1710 |
| **Confidence interval of slope (CI) ^b^** | 762.60 – 765.00 | 735.60 – 738.90 | 746.00 – 751.80 | 27700 - 27788 | 48600 - 48784 | 5980 - 6068 |
| **Confidence interval of intercept (CI) ^c^** | - 409.20 – - 389.00 | 476.80 – 502.40 | -546.90 –  - 490.90 | - 4120 -  - 3766 | - 4800 -  - 4466 | - 1900 -  - 1520 |
| **Relative uncertainty (u)** | 0.0059 (0.59%) | 0.0041  (0.41%) | 0.0656  (6.56%) | 0.0080  (0.80%) | 0.0032  (0.32%) | 0.0719  (7.19%) |
| **Expanded uncertainty (u, 95%) ^d^** | 0.0118 (1.18%) | 0.0082 (0.82%) | 0.1312 (13.12%) | 0.0160  (1.60%) | 0.0065  (0.65%) | 0.1439  (14.39%) |

**Table S6:** Statistical treatment of regression parameters via confidence intervals and uncertainty.

^a^ Regression equation: *A* = *a* + *bc*, where ‘A’ is the average peak area and ‘c’ is the concentration.

**^b^** Confidence interval of slope at confidence level 95%.

**^c^** Confidence interval of intercept at confidence level 95%.

**^d^** Expanded uncertainty at confidence level 95%.

**Table S7:**  Applicability and greenness assessment of the proposed HPTLC-densitometric, HPLC-DAD, and official methods using CACI, BAGI, AGSA, and CaFRI tools.

|  | **CACI tool [41]** | **BAGI tool [42]** | **AGSA tool [50]** | **CaFRI tool [51]** |
| --- | --- | --- | --- | --- |
| **Proposed HPTLC-densitometric method** | 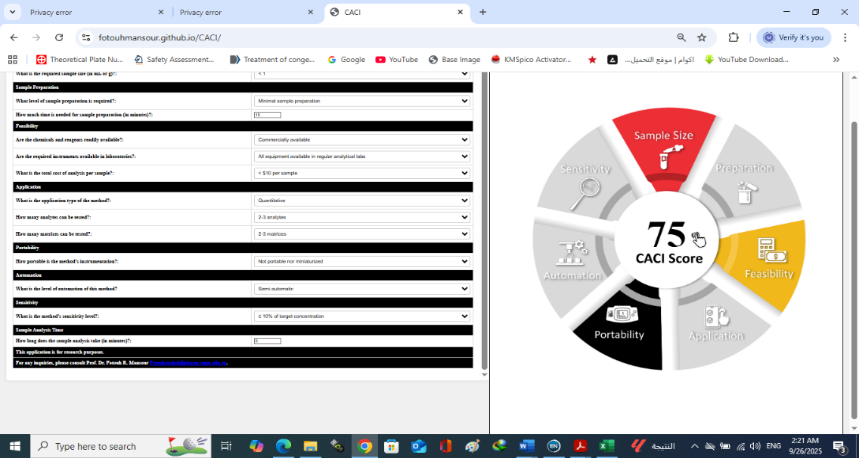 | 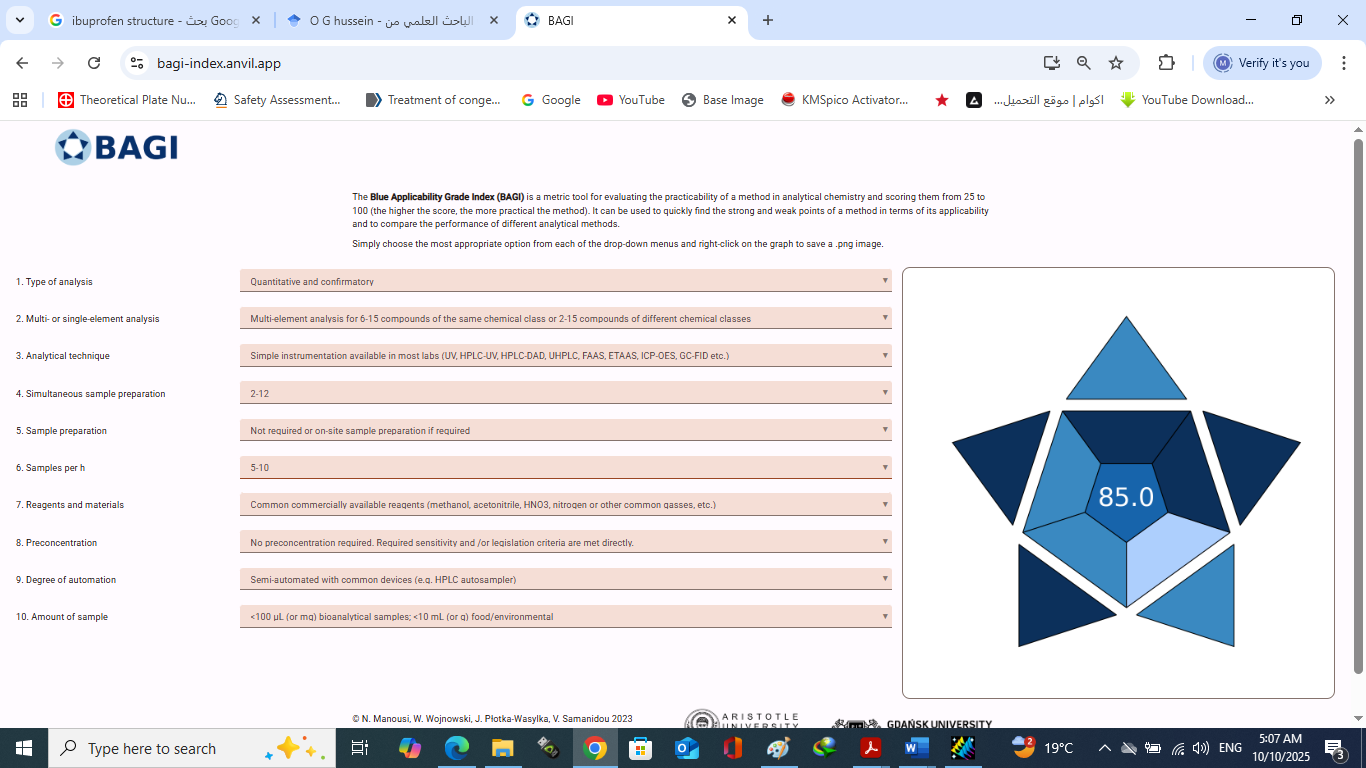 | 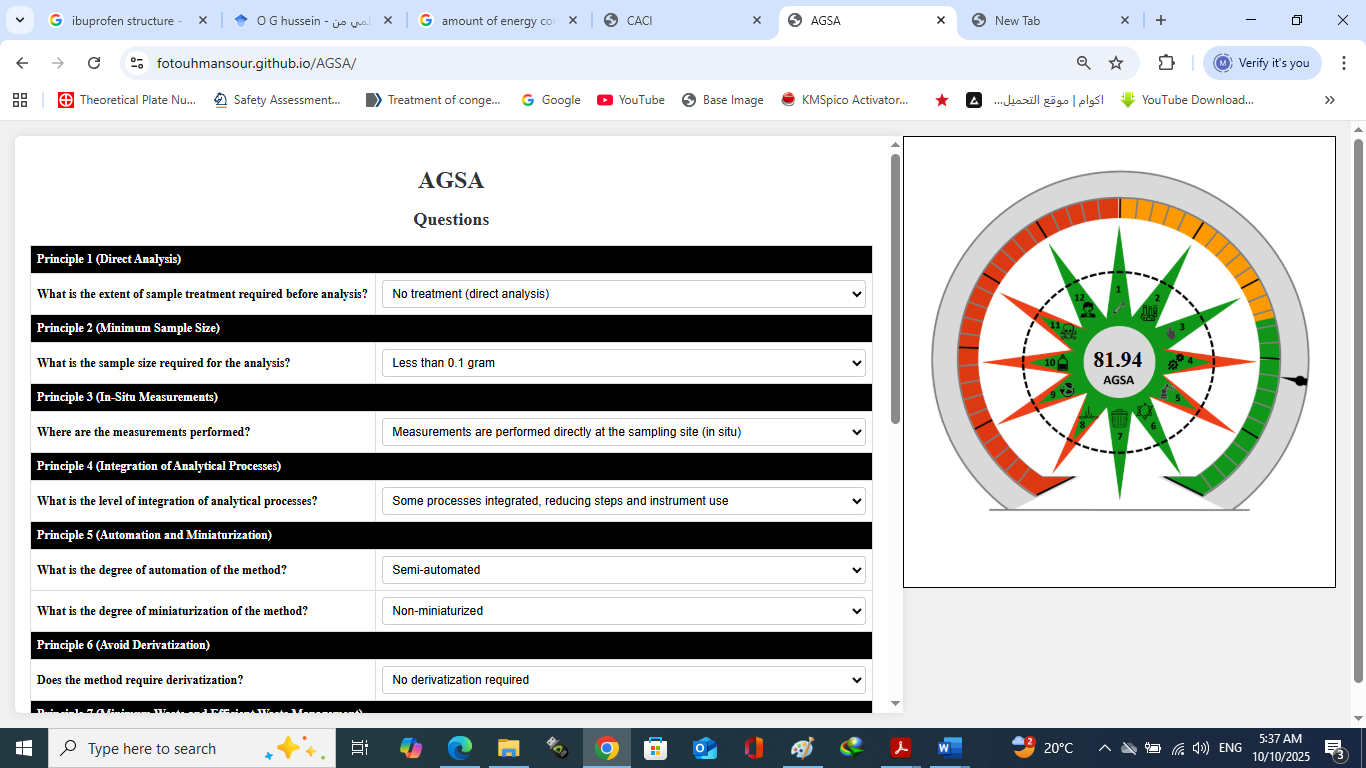 | 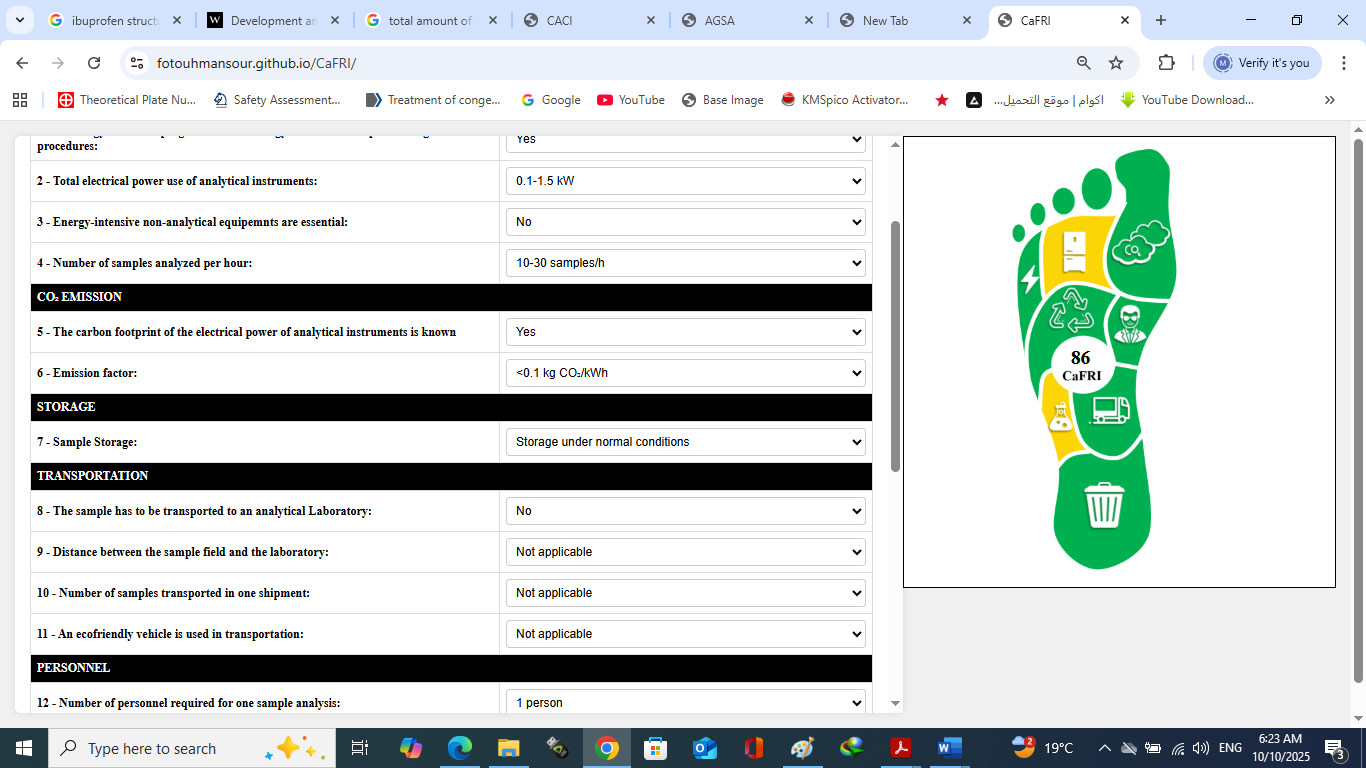 |
| **Proposed HPLC- DAD method** | 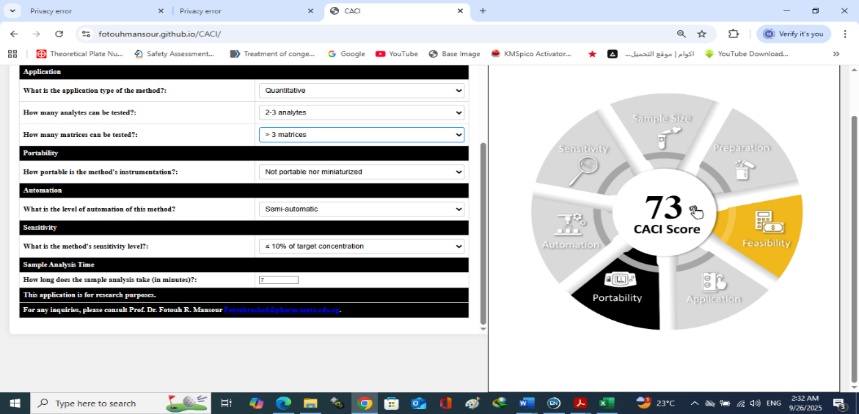 | 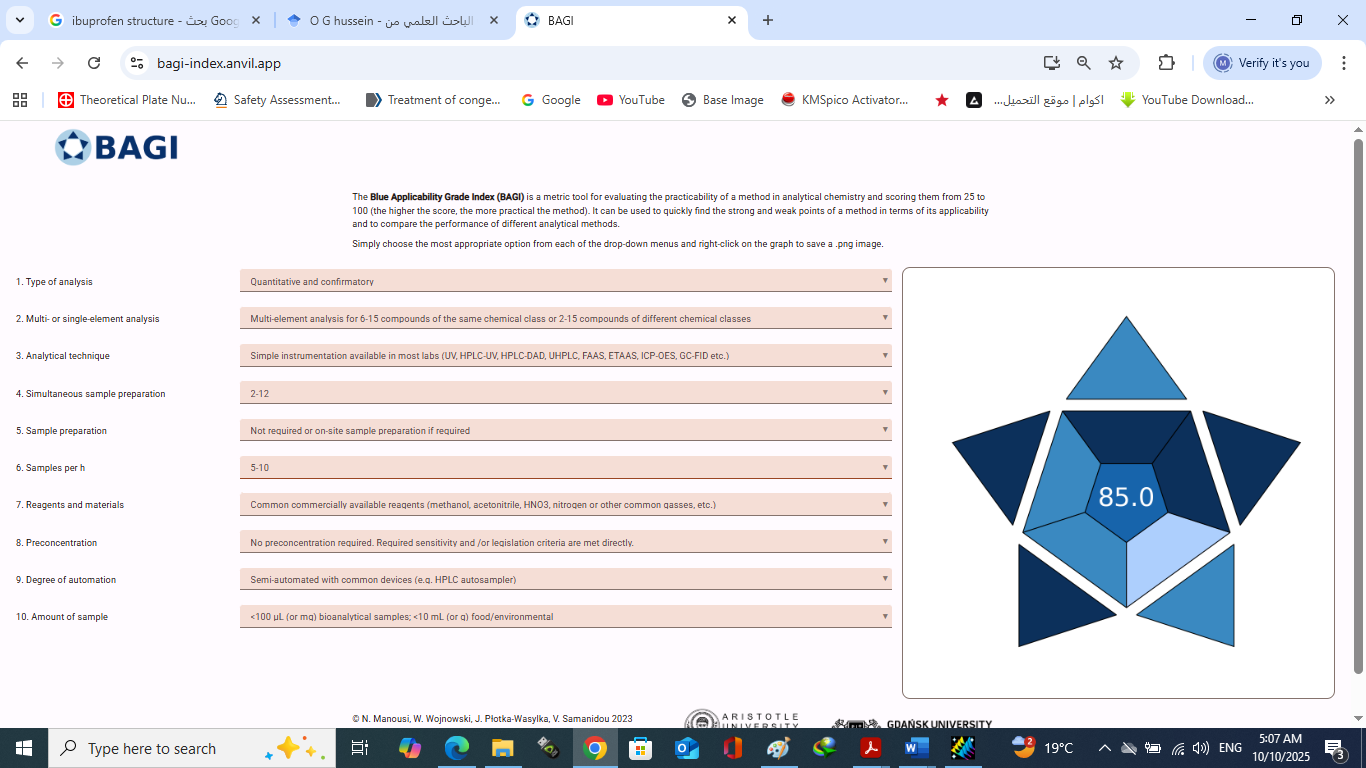 | 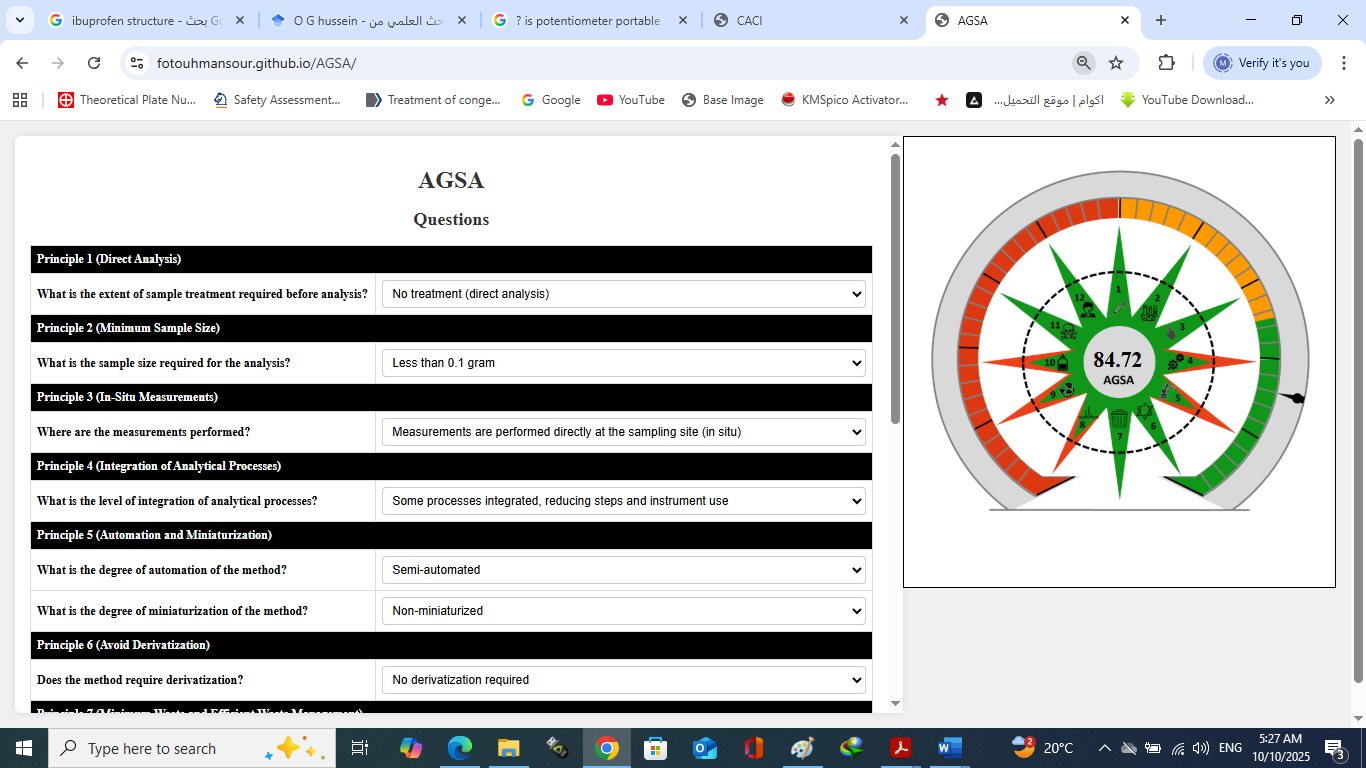 | 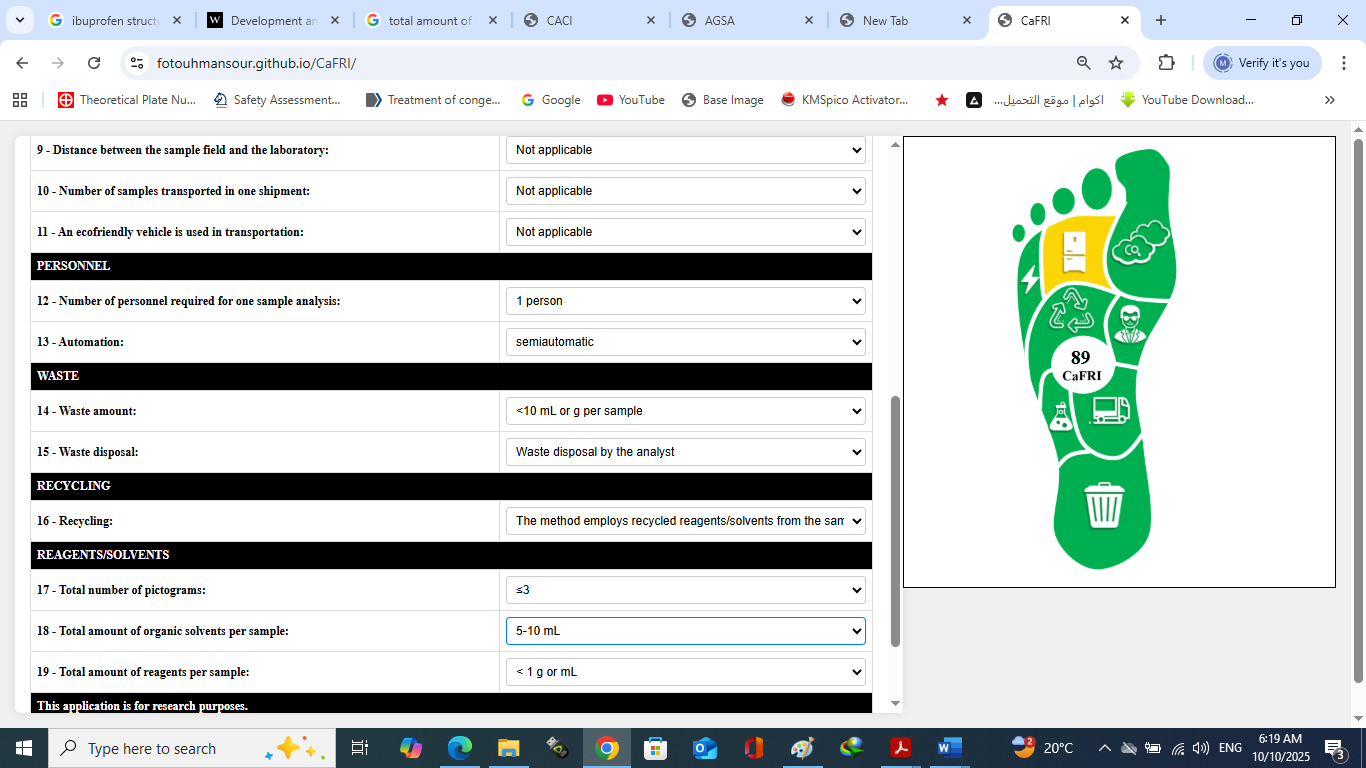 |
| **PHE Official method ^a^ [39]** | 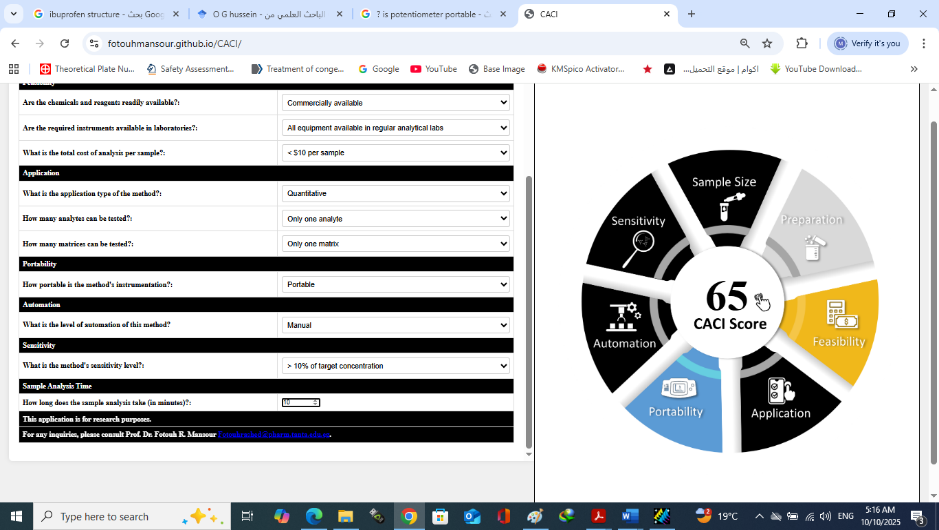 | 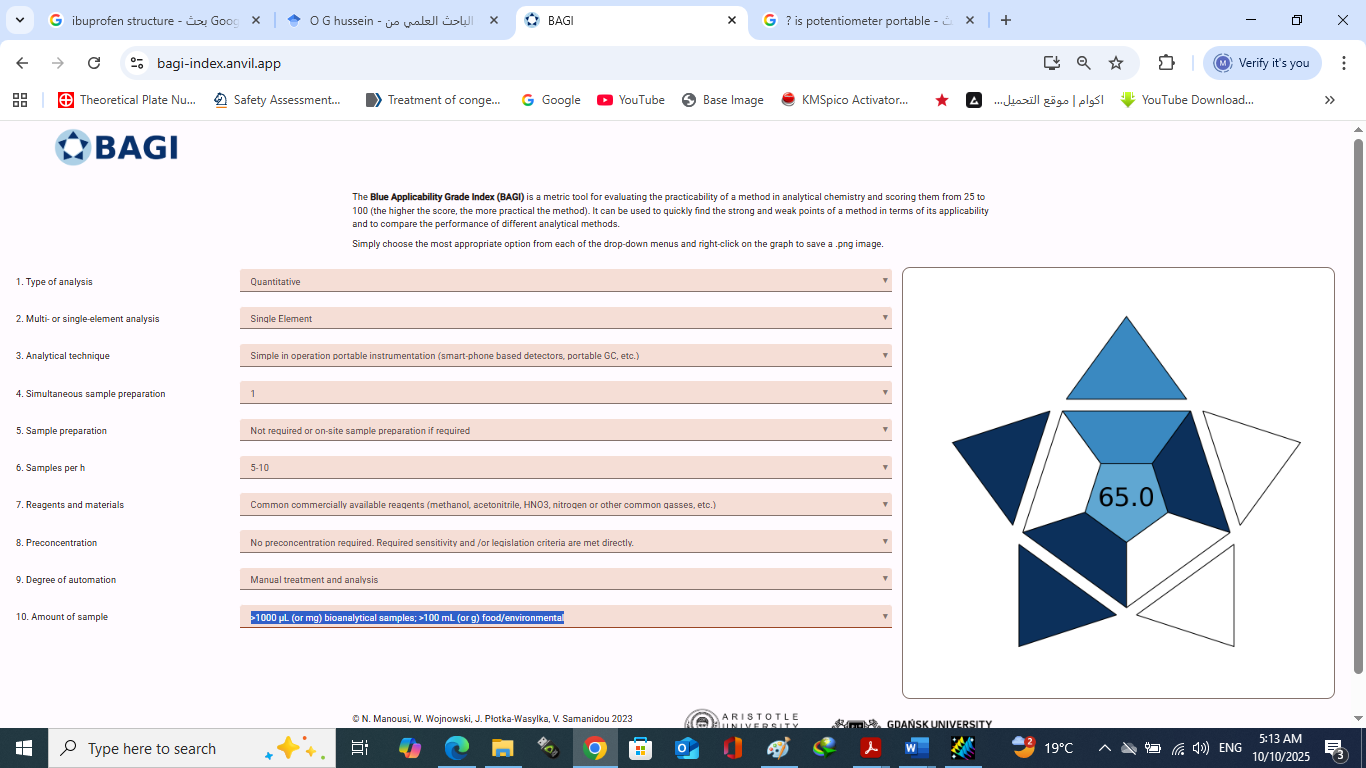 | 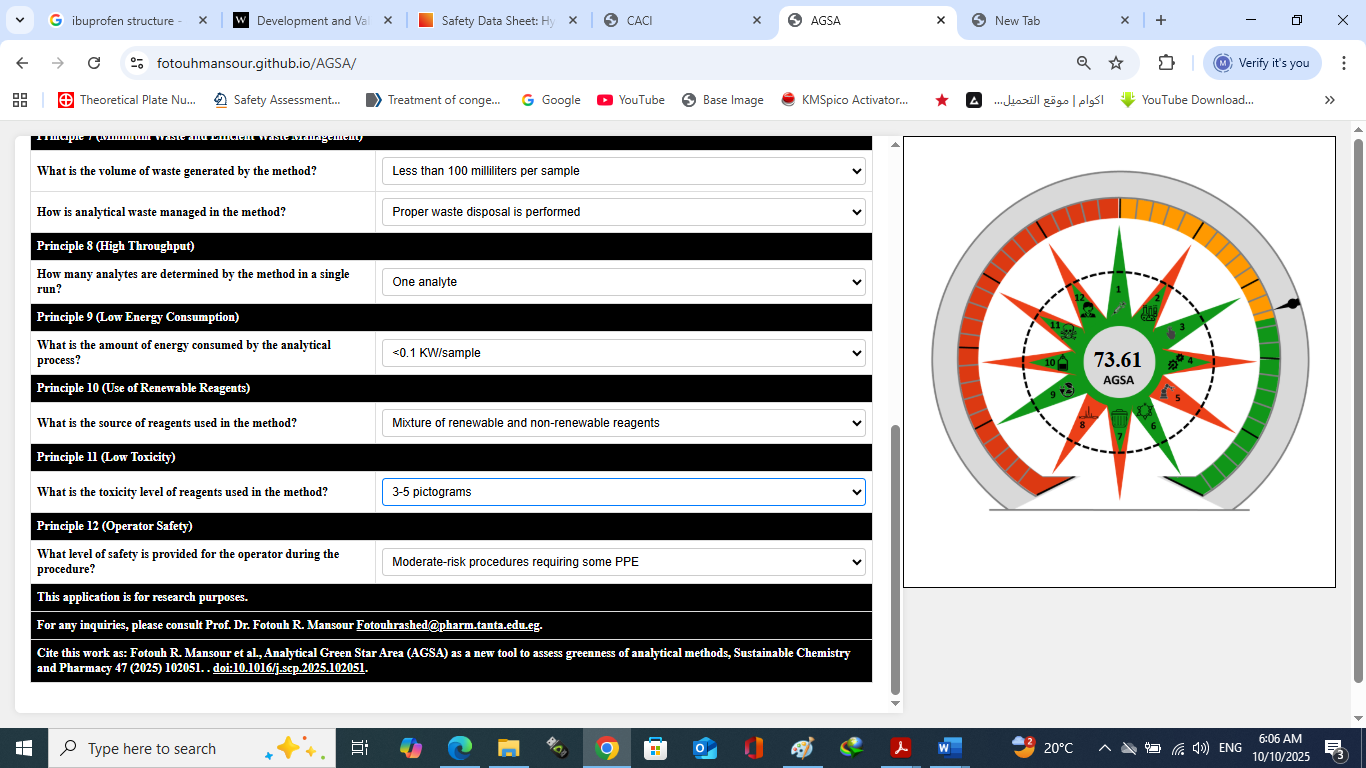 | 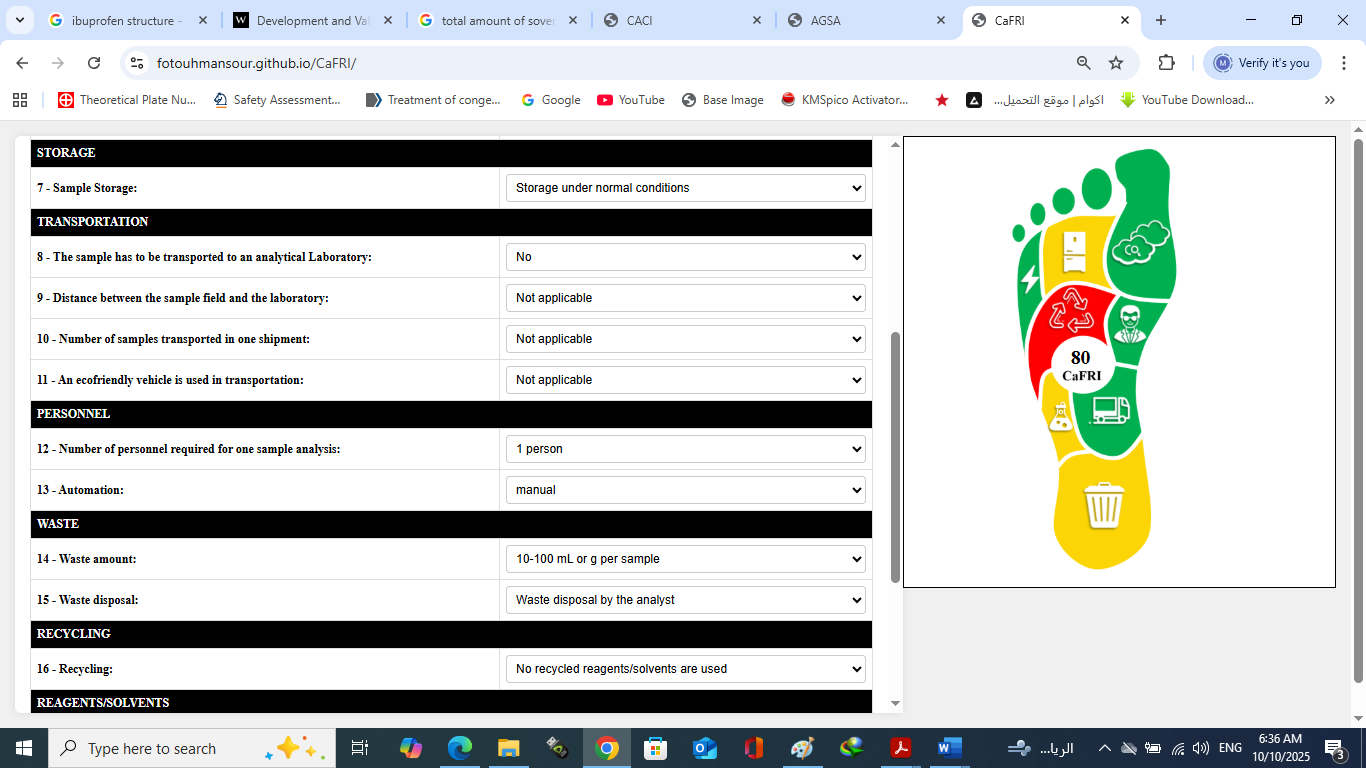 |
| **CPM Official method ^b^ [39]** | 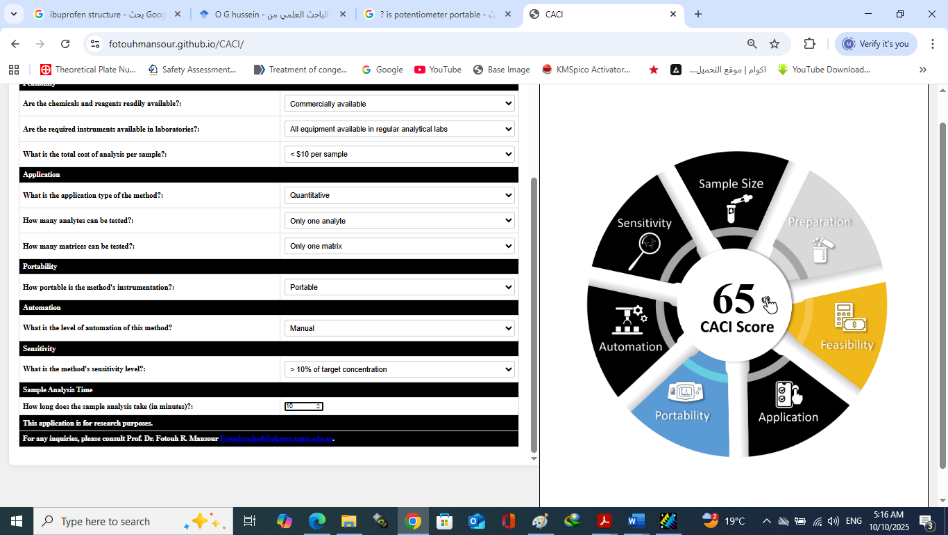 | 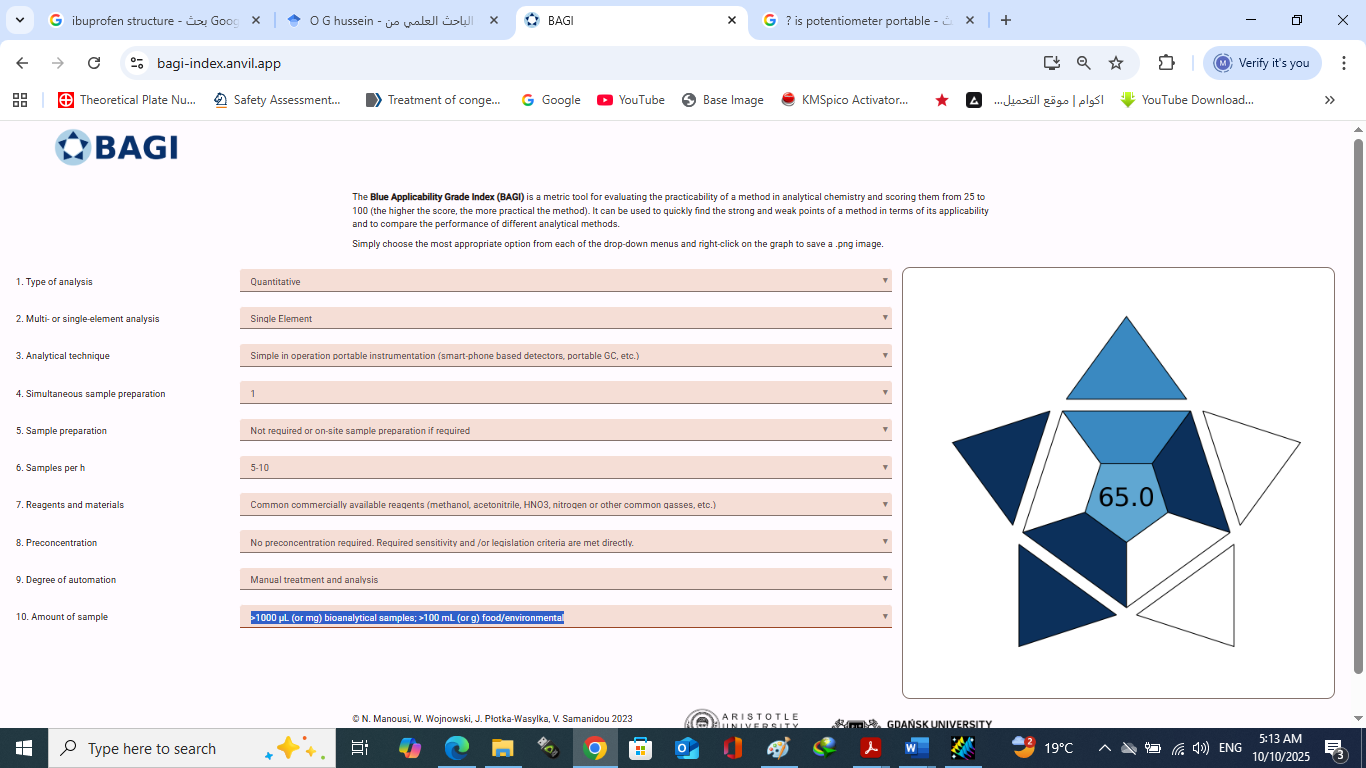 | 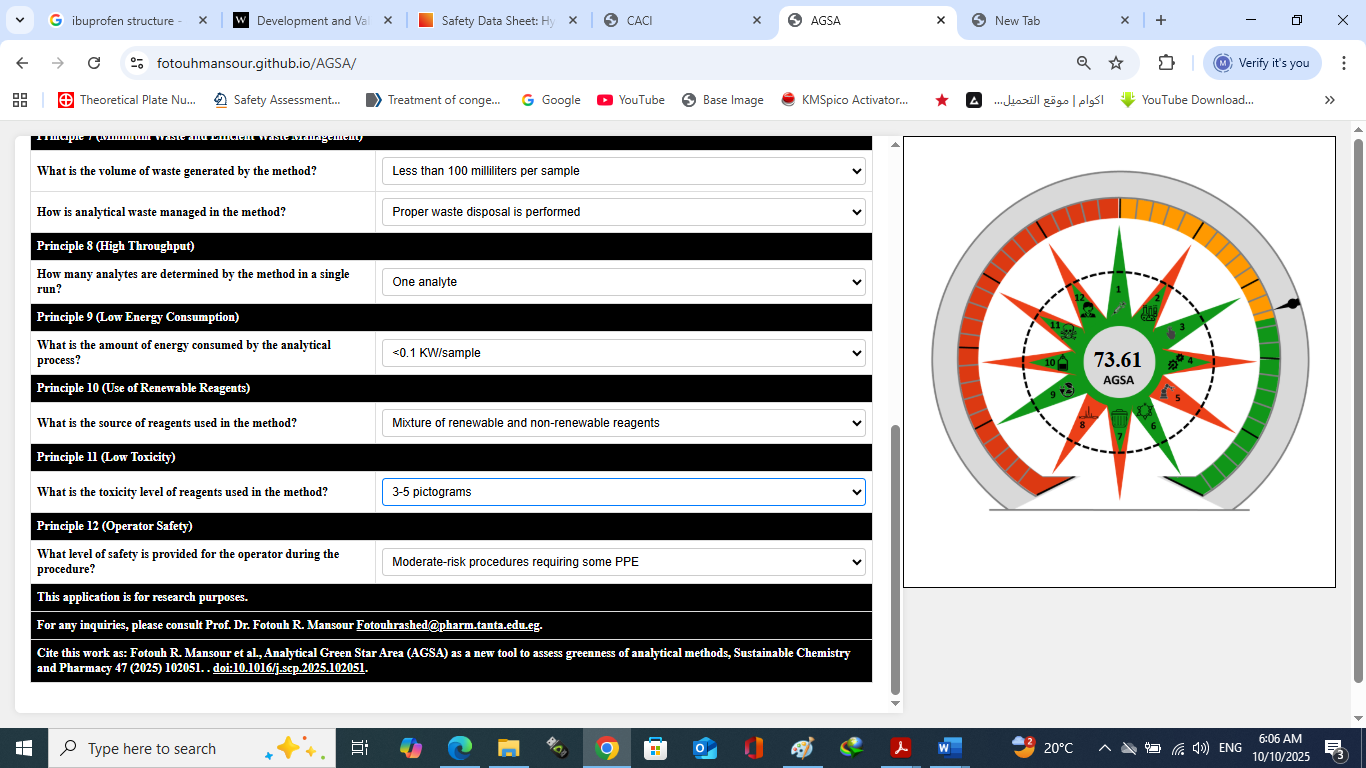 | 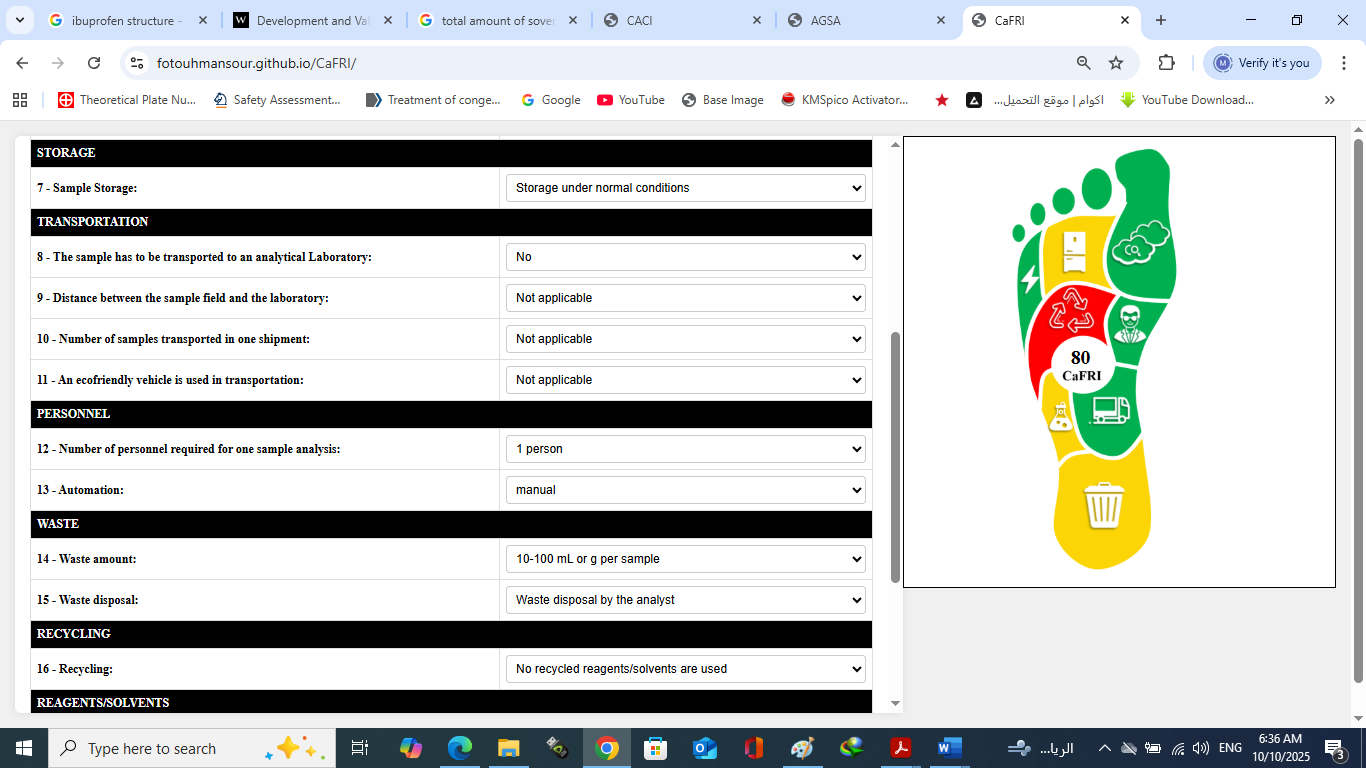 |
| **IBU Official method ^c^ [39]** | 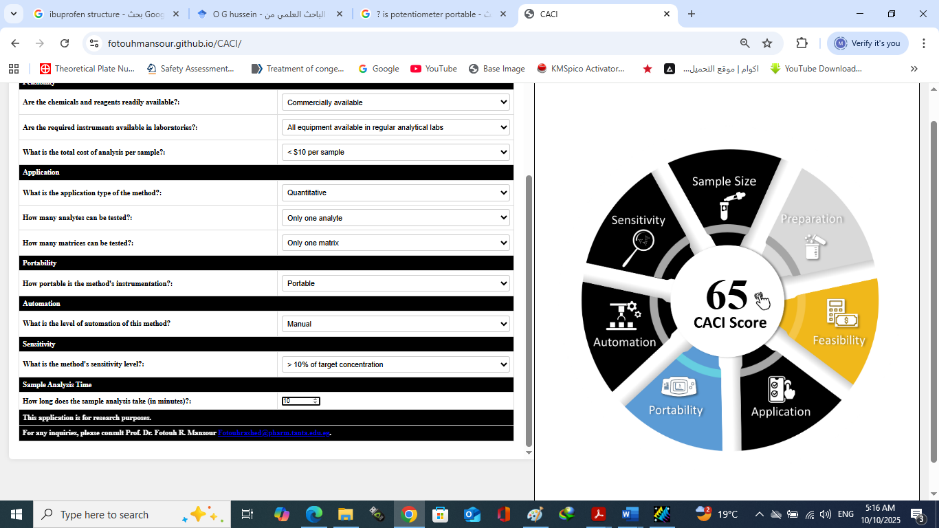 | 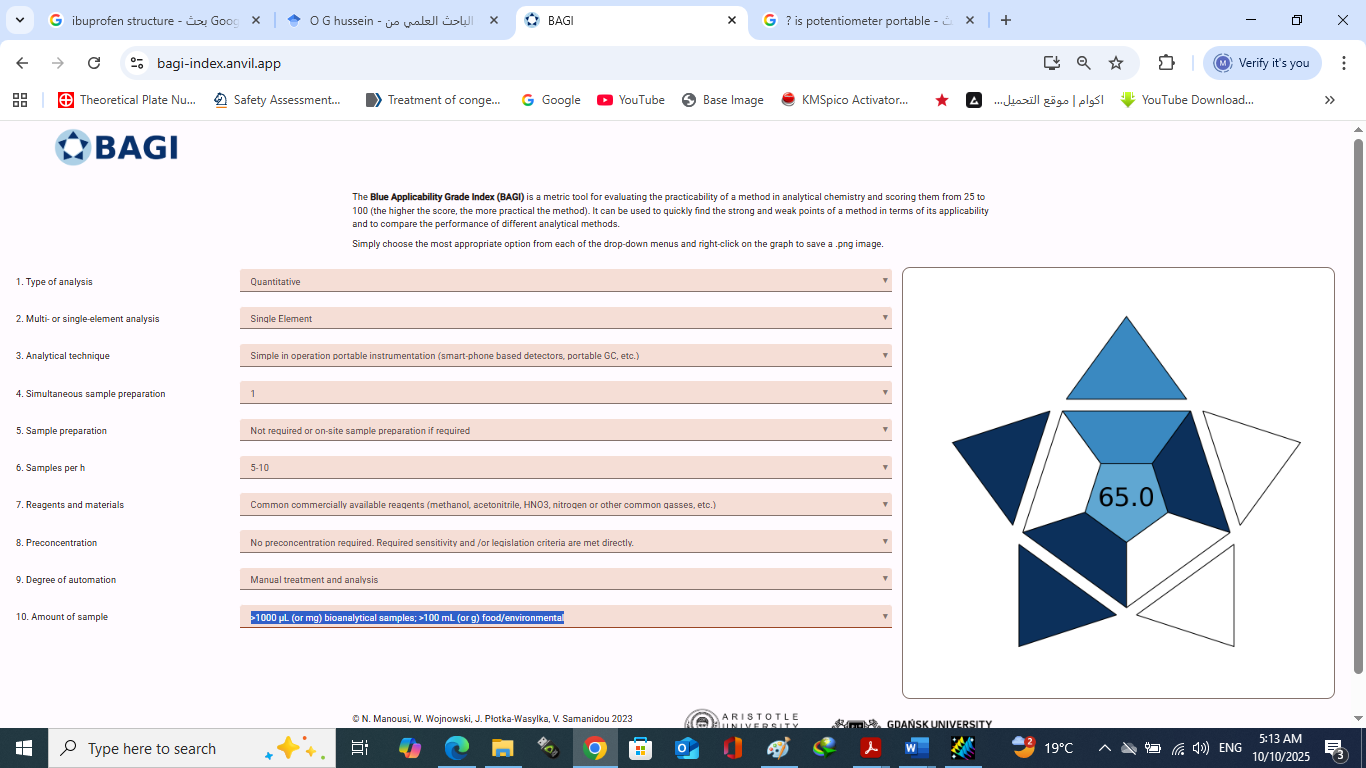 | 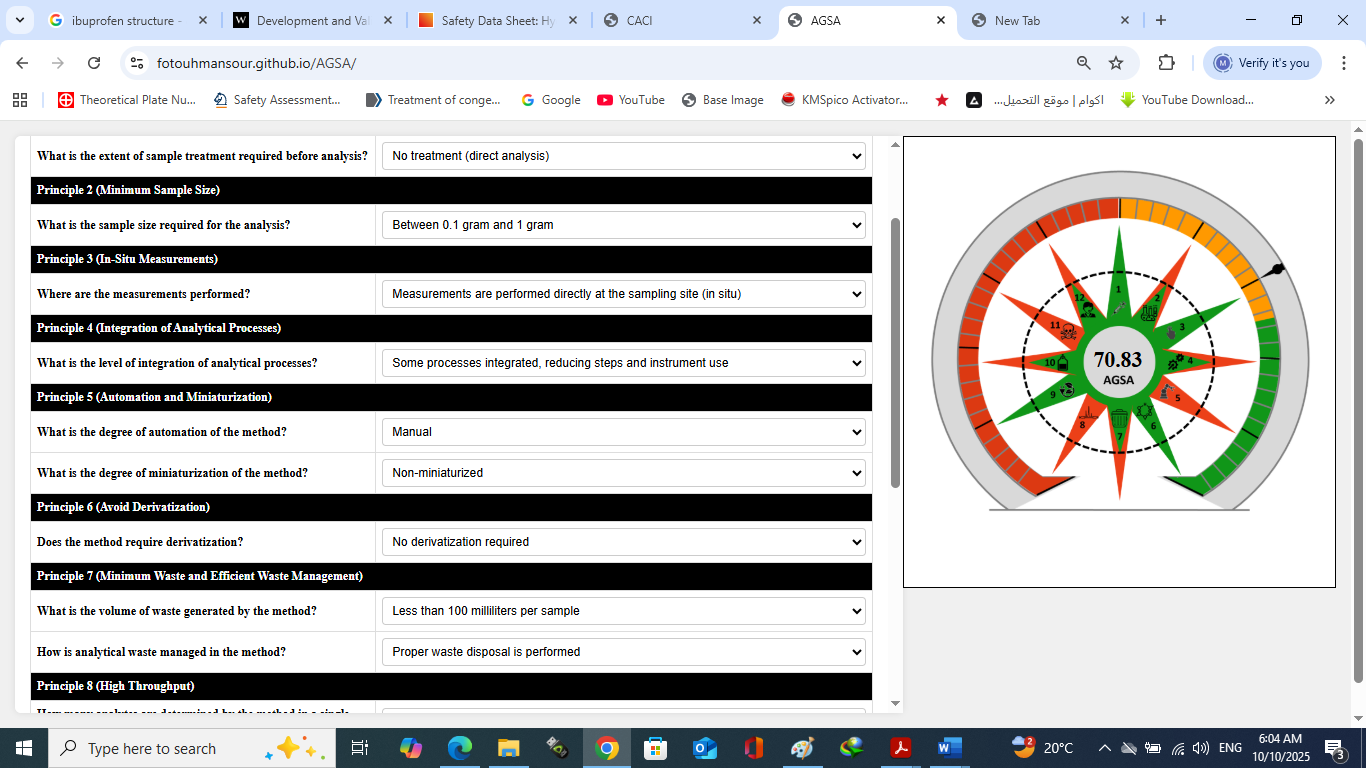 | 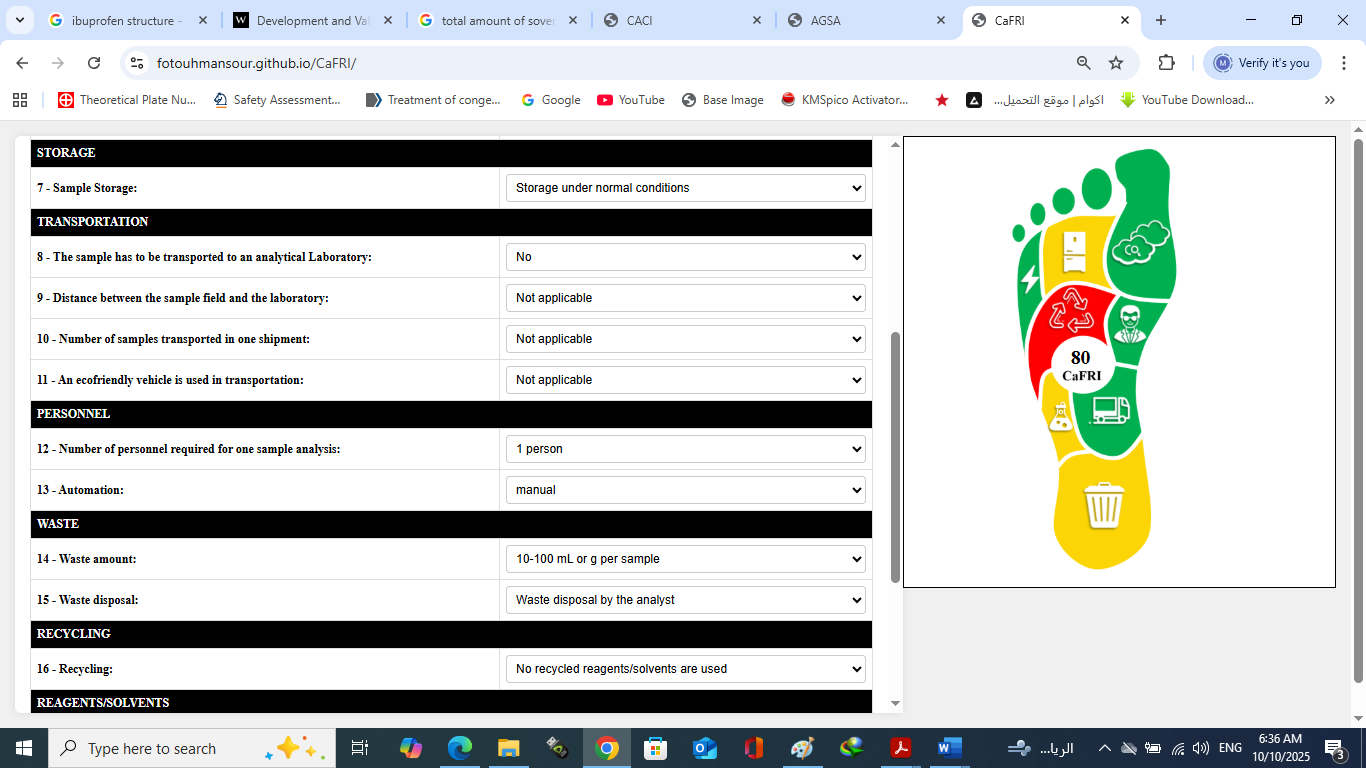 |

^a^ For Phenylephrine: Potentiometric titration method using ethanolic sodium hydroxide as a titrant.

^b^ For Chlorpheniramine: Potentiometric titration method using 0.1 M perchloric acid as a titrant.

^c^ For Ibuprofen: Titrimetric method using 0.1 M sodium hydroxide as a titrant.

**Graphical Abstract**


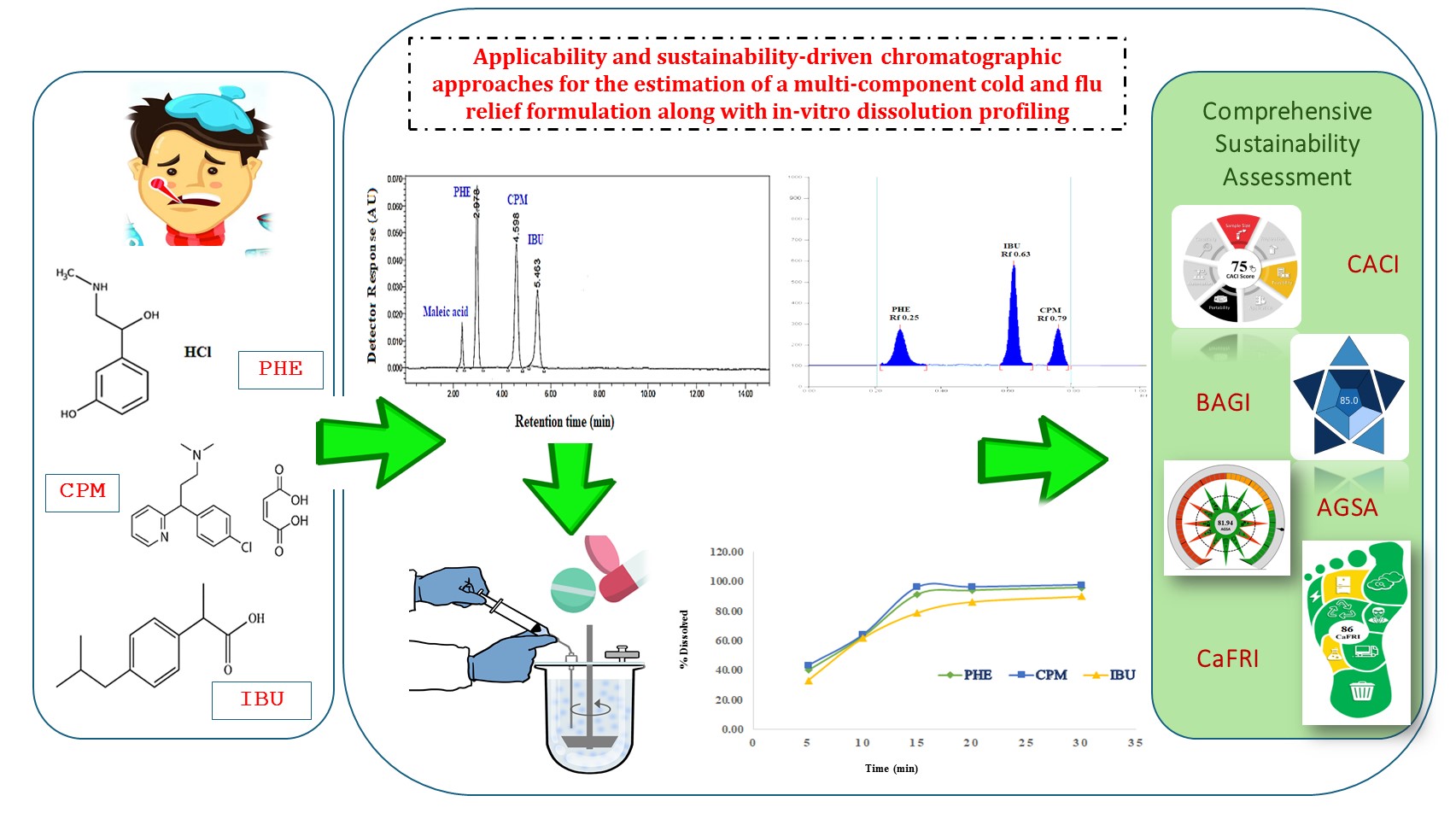

Supplement: Supplementary file 1 — Supplementary Material 1 [file 41598_2026_55497_MOESM1_ESM.docx]
